# Entangled photons enabled time-frequency-resolved coherent Raman spectroscopy and applications to electronic coherences at femtosecond scale

**DOI:** 10.1038/s41377-022-00953-y

**Published:** 2022-09-14

**Authors:** Zhedong Zhang, Tao Peng, Xiaoyu Nie, Girish S. Agarwal, Marlan O. Scully

**Affiliations:** 1 Department of Physics, City University of Hong Kong, Kowloon, Hong Kong SAR China; 2 City University of Hong Kong, Shenzhen Research Institute, Shenzhen, Guangdong 518057 China; 3 Institute for Quantum Science and Engineering, Texas A&M University, College Station, TX 77843 USA; 4 School of Physics, Xi’an Jiaotong University, Xi’an, Shaanxi 710049 China; 5 Department of Biological and Agricultural Engineering, Texas A&M University, College Station, TX 77843 USA; 6 Baylor University, Waco, TX 76704 USA

**Keywords:** Quantum optics, Ultrafast photonics, Nonlinear optics

## Abstract

Quantum entanglement has emerged as a great resource for spectroscopy and its importance in two-photon spectrum and microscopy has been demonstrated. Current studies focus on the two-photon absorption, whereas the Raman spectroscopy with quantum entanglement still remains elusive, with outstanding issues of temporal and spectral resolutions. Here we study the new capabilities provided by entangled photons in coherent Raman spectroscopy. An ultrafast frequency-resolved Raman spectroscopy with entangled photons is developed for condensed-phase molecules, to probe the electronic and vibrational coherences. Using quantum correlation between the photons, the signal shows the capability of both temporal and spectral resolutions not accessible by either classical pulses or the fields without entanglement. We develop a microscopic theory for this Raman spectroscopy, revealing the electronic coherence dynamics even at timescale of 50fs. This suggests new paradigms of optical signals and spectroscopy, with potential to push detection below standard quantum limit.

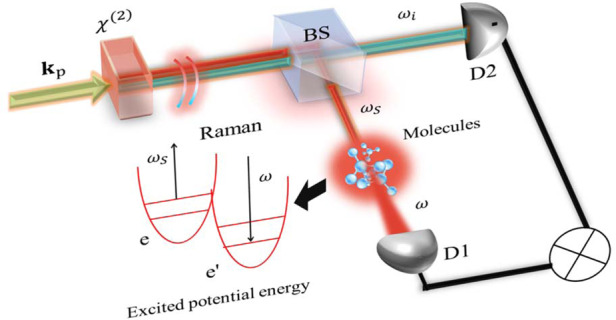

## Introduction

Coherent Raman spectroscopy, such as coherent anti-Stokes Raman scattering and stimulated Raman scattering, is a powerful tool for studying molecular vibrations^1,2^. It provides chemical selectivity in a noninvasive manner and has a significant penetration depth in tissues. Compared to spontaneous Raman signal, it is orders of magnitude more efficient, resulting in much faster remote sensing and detection^3^. It has found applications in various fields including probing time-resolved photochemical processes, medical imaging, and chemical sensing, providing a high degree of sensitivity and specificity. However, these approaches do have some drawbacks and limitations. In particular, there is always a trade-off between temporal and spectral resolutions.

Over last few decades quantum entanglement has emerged to have considerable applications in testing fundamental laws of physics^4–12^, as well as revolutionizing photonic applications, e.g., quantum imaging^13^, metrology^14,15^, sensing^16,17^, quantum computing^18,19^. It holds promise for future quantum technologies. The field of spectroscopy especially the nonlinear spectroscopy can benefit enormously by using entangled photons and more generally quantum light. The role of quantum entanglement in the context of two-photon spectroscopy was realized rather early. Fei et al. demonstrated entanglement-induced transparency in two-photon processes^20^. The occurrence of simultaneous excitation in two independent atoms due to entangled photons have been demonstrated in recent experiments^21,22^. In general the entangled photons have demonstrated to be beneficial in the two-photon absorption scheme^23,24^, and has incredible power in developing new spectroscopic techniques for complex materials^25–28^. Thanks to the technical advance in pulse shaping, the two-photon absorption with photon entanglement has been demonstrated in various experiments, incredibly suppressing the intermediate state relaxation that yields the optimal efficiency of populating higher excited states^29–33^. The entangled states of photons provide extra control knobs for selective molecular relaxation and radiative processes. The related signals may be extended into the sub-picosecond time-resolved regime, resulting in an ultrafast optical technique to probe the dynamics of various excitations. Recent work on stimulated Raman and pump-probe spectroscopies showed the enhanced resolution via photon entanglement through the model calculations^25,34,35^.

Because of the new parameter spaces with the entangled states of photons, especially the temporal and frequency correlations, the desired population relaxation, and radiative processes are accessible. The ultrafast dynamics of electronic coherence is fundamentally important for reaction kinetics and internal conversions. However, it is a challenging task due to its rapid decay^36–41^. Li et al. proposed recently a quantum simulation scheme using circuit representation, to capture quantum dissipative dynamics^42^. Nevertheless, quantum states of light may be powerful for exploiting the ultrafast electronic processes in semiconductor materials. This has been indicated from the semiconductor quantum-light sources taking advantage of generating high photon flux and the scalability^43,44^. More delicate information about the structure and dynamics in nanostructures and low-dimensional materials have to be read out by ultrafast coupling to photon states^45–51^. Subsequent studies aiming at ultrafast multi-photon coincidence counting spectrum revealed the highly-resolved excitonic relaxation pathways and achieved super-resolved imaging by manipulating the temporal and spectral profiles not accessible by classical pulses^52–55^. It turns out that the unusual band spectrum of entangled photons, which has been shown to achieve superresolution and to beat standard quantum limit, renders the quantum-light spectroscopy as a new promising route towards novel applications in ultrafast and ultra-sensitive tomography^56–60^.

In this work, we demonstrate new capabilities brought out by the use of entangled photons in femtosecond time-resolved coherent Raman spectroscopy to monitor the electronic and vibrational coherences. This leads to the QFRS (Quantum femtosecond Raman spectroscopy), providing a scheme with photon entanglement and nonlinear interferometry that reside in a different category from the CARS with classical laser pulses and femtosecond stimulated Raman scattering (FSRS)^1–3,34,37^. Specifically, we show the time-frequency resolutions obtained by the photon entanglement. Explicit results are presented for a quantum extension of FAST CARS (Femtosecond adaptive spectroscopic technique for coherent anti-Stokes Raman scattering)^3,61,62^.

## Results

### Quantum FAST CARS: vibrational coherence in electronic ground state

**Fig. 1:**
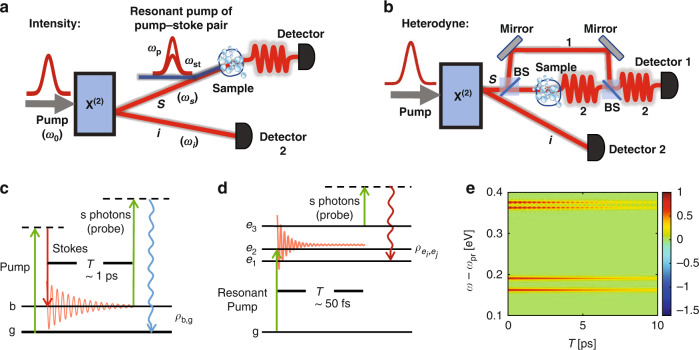
**a** Schematic of entangled twin photons as ultrafast probe for molecules, where the parametric down conversion through a beta barium borate (BBO) crystal and multi-photon detection are presented. **b** Quantum heterodyne scheme of detection where a local oscillator interferes with emitted photons in *s* arm; Detector 1 records the interference term whereas detector 2 records the idler photon number, yielding the joint detection. **c** Level scheme of microscopic model in Quantum FAST CARS. **d** Level scheme of microscopic model in QFRS for electronically excited states. **e** Quantum FAST CARS signal from Eq. (6), taking the 4 Raman-active modes A_1_, E and T_2_ in methane (CH_4_), where the parameters are ${\sigma }_{0}^{-1}=35$ fs for broadband probe and ${\gamma }_{bg}^{-1}=16$ps, 20ps, 15ps, 22ps

To start off, we consider the FAST CARS with entangled photons, where the molecules are driven by a pair of classical pulses. After a time delay, the beam in *s* arm of the entangled twin photons serves as a Raman probe off-resonantly interacting with molecules, whereas the idler beam propagates freely and provides a reference, as depicted in Fig. 1a. Referring to the scheme in Fig. 1c, the field-molecule interaction of Raman process is

1 $$V(t)=\mathop{\sum }\limits_{j=1}^{N}\mathop{\sum}\limits_{b}{\alpha }_{bg}^{(j)}\left|b\right\rangle {\left\langle g\right|}_{j}(t){E}_{s}(t){E}_{s}^{{\dagger} }(t)+{{{\rm{h.c.}}}}$$

where ${\alpha }_{bg}^{(j)}$ is the Raman polarizability and the field *E*_*s*_(*t*) contains multiple frequency modes (Supplementary section S1). Usually $\left|b\right\rangle {\left\langle g\right|}_{j}(t)=\left|b\right\rangle {\left\langle g\right|}_{j}{e}^{i({\omega }_{b}-{\omega }_{g})t}$ for closed systems but we will not adopt this assumption hereafter, in order to involve more general cases whose dynamics may be described by the reduced density matrix. *N* denotes the number of molecules. $\left|{{\Psi }}\right\rangle =\int\nolimits_{-\infty }^{\infty }\int\nolimits_{-\infty }^{\infty }{{{\rm{d}}}}{\omega }_{s}{{{\rm{d}}}}{\omega }_{i}\,{{\Phi }}({\omega }_{s},{\omega }_{i}){a}_{{\omega }_{s}}^{{\dagger} }{a}_{{\omega }_{i}}^{{\dagger} }\left|0\right\rangle$ and

2 $${{\Phi }}({\omega }_{s},{\omega }_{i})={E}_{0}({\omega }_{s}+{\omega }_{i}){{{\rm{sinc}}}}\left[\frac{{{\Delta }}k({\omega }_{s},{\omega }_{i})L}{2}\right]{e}^{i{{\Delta }}k({\omega }_{s},{\omega }_{i})L/2}$$

is the wave function for the entangled twin photons with ${{\Delta }}k({\omega }_{s},{\omega }_{i})L=({\omega }_{s}-\frac{{\omega }_{0}}{2}){T}_{s}+({\omega }_{i}-\frac{{\omega }_{0}}{2}){T}_{i}$ where *T*_*s*_(*T*_*i*_) is the time delay between the photons in *s* (reference) arm and the SPDC pump field, due to the group velocity dispersion in the nonlinear crystal. *E*_0_ is the pump driving SPDC process, and *ω*_0_/2 is the central frequency of the downconverted beams.

As the anti-Stokes shift is of the most interest, the Heisenberg equation of motion for emitted photons is found to be

3 $${\dot{a}}_{s,\omega }=\frac{1}{2\pi i}\mathop{\sum }\limits_{j=1}^{N}\mathop{\sum}\limits_{b}{\alpha }_{bg}^{(j),* }{{{{\mathcal{E}}}}}_{s,\omega }\left|g\right\rangle {\left\langle b\right|}_{j}(t){E}_{s}(t){e}^{i\omega (t-T)}$$

where ${E}_{s}(t)=\frac{1}{2\pi }{\sum }_{v}{E}_{s}(v){e}^{-iv(t-T)}$ and ${E}_{s}(v)={{{{\mathcal{E}}}}}_{s,v}{a}_{s,v}$ is the Fourier component of the electric field in s arm. ${{{{\mathcal{E}}}}}_{s,v}$ is a constant having dimension of electric field, which is subject to the beam power across unit area, i.e., the Poynting vector $| {{{\bf{S}}}}| =2\pi c{\sum }_{v}| {{{{\mathcal{E}}}}}_{s,v}{| }^{2}$. $[{a}_{s,v},{a}_{s,v^{\prime} }^{{\dagger} }]={\delta }_{v,v^{\prime} }$. *T* is the time delay of *s*-arm photons relative to the pump pulse. Using the perturbation expansion against Eq. (1), we solve for Eq. (3)

4 $${a}_{s,\omega }=\frac{1}{2\pi i}\mathop{\sum }\limits_{j=1}^{N}\mathop{\sum}\limits_{b}{\alpha }_{bg}^{(j),* }{{{{\mathcal{E}}}}}_{s,\omega }\int\nolimits_{-\infty }^{t}\left|g\right\rangle {\left\langle b\right|}_{j}(\tau ){E}_{s}(\tau ){e}^{i\omega (\tau -T)}d\tau$$

and thus find the four-point field correlation function playing a key role in understanding the unusual spectral properties of Raman signals with photon entanglement. A joint detection of the spectral-resolved transmissions in two arms gives the intensity-correlated signal $S(\omega ,{\omega }_{i};T)={\langle {E}_{s}^{{\dagger} }(\omega ){E}_{i}^{{\dagger} }({\omega }_{i}){E}_{i}({\omega }_{i}){E}_{s}(\omega )\rangle }_{\rho }$ where *E*_*i*_(*ω*_*i*_) denotes the Fourier component of the electric field in reference arm. This resides in a different category from the paradigm in Ref. ^3^. Inserting Eq. (4) into the signal, some algebra gives

5 $$\begin{array}{ll}S(\omega ,{\omega }_{i};T)=\,\frac{| {{{{\mathcal{E}}}}}_{s,\omega }{| }^{4}}{4{\pi }^{2}}\mathop{\sum}\limits_{b,b^{\prime} }\mathop{\sum}\limits_{j\ne j^{\prime} }{\alpha }_{b^{\prime} g}^{(j^{\prime} )}{\alpha }_{bg}^{(j),* }{\iint }_{-\infty }^{t}d\tau d\tau ^{\prime} {\rho }_{b^{\prime} g}^{(j^{\prime} ),* }(\tau ^{\prime} )\\ \qquad\qquad\quad\times\, {\rho }_{bg}^{(j)}(\tau ){e}^{-i\omega (\tau ^{\prime} -\tau )}\left\langle {{\Phi }}\right|{E}_{s}^{{\dagger} }(\tau ^{\prime} ){E}_{s}(\tau ){E}_{i}^{{\dagger} }({\omega }_{i}){E}_{i}({\omega }_{i})\left|{{\Phi }}\right\rangle \end{array}$$

where Φ refers to the two-photon wave function. We included in Eq. (5) the most significant terms whereas the spontaneous Raman emission has been dropped. Eq. (5) provides the most general formalism for the coherent Raman signal with quantum light and nonlinear interferometry.

We proceed via inserting Eq. (2) into Eq. (5) and evaluating the field correlation function, where the identical molecules are assumed so that ${\alpha }_{bg}^{(j)}={\alpha }_{bg}$. A lengthy but straightforward algebra gives rise to the Quantum FAST CARS signal

6 $$\begin{array}{ll}{S}_{{{{\rm{QV}}}}}(\omega ,{\omega}_{i};T)=\,\frac{N(N-1)}{8{\pi}^{2}}| {{{{\mathcal{E}}}}}_{s,\omega }{|}^{6}| {{{{\mathcal{E}}}}}_{i,{\omega}_{i}}{|}^{2}\left|\right.{\sum}_{b}{\alpha}_{bg}^{*}{\rho}_{bg}(T)\\ \qquad\qquad\qquad\times\, {{\Phi}}(\omega -{\omega}_{bg}-i{\gamma}_{bg},{\omega}_{i}){\left|\right.}^{2}\end{array}$$

with ${\rho }_{bg}(t)={\rho }_{bg}{e}^{-(i{\omega }_{bg}+{\gamma }_{bg})t}$ where ${\gamma }_{bg}^{-1}$ quantifies the dephasing of the vibrational coherence. Besides the enhanced signal intensity via coherence, Eq. (6) indicates the spectral resolution governed by photon entanglement that contains new control knobs such as photon arrival time and bandwidth.

For a neat understanding of the signal, we focus on the optimal photon entanglement, i.e., *T*_*s*_ = *T*_*i*_. The two-photon amplitude in Eq. (2) indicates a Raman resonance *ω* − *ω*_pr_ = *ω*_*bg*_ defining the probe frequency *ω*_pr_ = *ω*_0_ − *ω*_*i*_. This leads to the spectral resolution ${T}_{s}^{-1}$, which reveals a considerable enhancement not accessible by classically shaped pulses. To make this clear, we calculate the Raman signals with two incoherent states of light: (i) Fock state of photons that is fully separable and (ii) the psuedo-thermal light ${\rho }_{{{{\rm{pht}}}}}=\iint {{{\rm{d}}}}v{{{\rm{d}}}}{v}_{i}| {{\Phi }}(v,{v}_{i}){| }^{2}\left|{1}_{s,v};{1}_{i,{v}_{i}}\right\rangle \left\langle {1}_{s,v};{1}_{i,{v}_{i}}\right|$ having classical correlation which has been used in information processing. For (i), the spectral resolution is governed by the single-photon amplitude ${{{\Phi }}}_{s}({\omega }_{s}-{\omega }_{0})\propto {e}^{-{({\omega }_{s}-{\omega }_{0})}^{2}/2{\sigma }_{0}^{2}}$ having the identical time duration ${\sigma }_{0}^{-1}$ as the pump pulse, as seen from Eq. 20 in section S1 of the Supplementary Information (SI). In CARS experiments, the conjugated time and frequency resolutions results in the challenge to distinguish the vibrational modes once $| {\omega }_{{b}_{1},{b}_{2}}| \ll {\sigma }_{0}$. Using photon entanglement, the spectral resolution can be controlled independently and can be therefore greatly enhanced such that ${T}_{s}^{-1} < | {\omega }_{{b}_{1},{b}_{2}}| \ll {\sigma }_{0}$, evident by Fig. 1e that keeps high temporal resolution. For (ii) as entailed by Eq. (19) in Supplementary section S1, the signal shows a high spectral resolution ${T}_{s}^{-1}$, whereas the temporal resolution is no longer there. This is expected from the random phase between photon pairs that results in arbitrary arrival time of photons.

### Femtosecond spectroscopy with entangled light: Intensity-correlated QFRS

To monitor the ultrafast dynamics of electronic coherence in excited states of molecules, the probe pulse has to act after a delay *T* following a resonant photoexcitation or a propagation of the excited states, depicted in Fig. 1d. The Raman signal is produced when the *s*-arm photon scatters off the excited-state coherence resulting in the electronic polarizability *α*(*t*) describing the Raman transitions between excited states. The general expression in Eq. (5) allows us to find the intensity-correlated Raman signal incorporating the *α*(*t*), so that

7 $$\begin{array}{ll}S(\omega ,{\omega }_{i};T)=\,\frac{| {{{{\mathcal{E}}}}}_{s,\omega }{| }^{4}}{4{\pi }^{2}}\mathop{\sum}\limits_{e^{\prime} ,e^{\prime\prime} }\mathop{\sum}\limits_{j\ne j^{\prime} }{\alpha }_{e^{\prime} ,e}^{(j)}{\alpha }_{e,e^{\prime\prime} }^{(j^{\prime} )}{\iint }_{-\infty }^{t}d\tau d\tau ^{\prime} {\rho }_{e,e^{\prime} }^{(j)}(\tau )\\ \qquad\qquad\quad\times\, {\rho }_{e,e^{\prime\prime} }^{(j^{\prime} ),* }(\tau ^{\prime} ){e}^{i\omega (\tau -\tau ^{\prime} )}\left\langle {{\Phi }}\right|{E}_{s}^{{\dagger} }(\tau ^{\prime} ){E}_{s}(\tau ){E}_{i}^{{\dagger} }({\omega }_{i}){E}_{i}({\omega }_{i})\left|{{\Phi }}\right\rangle \end{array}$$

with ${\alpha }_{e^{\prime} ,e}^{(j)}={\alpha }_{e,e^{\prime} }^{(j),* }$. Proceeding via the algebra similar as before, we find the QFRS signal (Supplementary sections S1 & S3)

8 $${S}_{{{{\rm{QE}}}}}(\omega ,{\omega }_{i};T)=\frac{N(N-1)}{32{\pi }^{4}}| {{{{\mathcal{E}}}}}_{s,\omega }{| }^{6}| {{{{\mathcal{E}}}}}_{i,{\omega }_{i}}{| }^{2}{\left|\mathop{\sum}\limits_{e^{\prime} }{\alpha }_{e,e^{\prime} }^{* }{f}_{e,e^{\prime} }(T)\right|}^{2}$$

where the Raman line-shape function is defined as

9 $${f}_{e,e^{\prime} }(T)=\int\nolimits_{-\infty }^{\infty }{{{\rm{d}}}}\tau \int\nolimits_{-\infty }^{\infty }{{{\rm{d}}}}\omega ^{\prime} {\rho }_{e,e^{\prime} }(\tau ){e}^{i(\omega -\omega ^{\prime} )(\tau -T)}{{\Phi }}(\omega ^{\prime} ,{\omega }_{i})$$

Eq. (9) indicates the role of the photon entanglement whose unusual band properties may provide versatile tool for controlling the ultrafast electron dynamics in molecules.

The electronically excited-state dynamics coupled to nuclear motions has to be considered. This may facilitate a quantitative understanding of the inhomogeneous dephasing in condensed-phase molecules. We adopt the Fröhlich–Holstein model for molecules

10 $$H={H}_{{{{\rm{vib}}}}}\left|g\right\rangle \left\langle g\right|+\mathop{\sum}\limits_{i}\left({\omega }_{{e}_{i},g}+{\lambda }_{s}^{(i),2}{v}_{s}+{H}_{{{{\rm{vib}}}}}^{i}\right)\left|{e}_{i}\right\rangle \left\langle {e}_{i}\right|$$

and ${H}_{{{{\rm{vib}}}}}={\sum }_{s}{v}_{s}{b}_{s}^{{\dagger} }{b}_{s}$ and ${H}_{{{{\rm{vib}}}}}^{i}={\sum }_{s}\left[{v}_{s}{b}_{s}^{{\dagger} }{b}_{s}-{\lambda }_{s}^{(i)}{v}_{s}({b}_{s}+{b}_{s}^{{\dagger} })\right]$, where *b*_*s*_ is the annihilation operator of the *s*-th vibration having the frequency *v*_*s*_. ${\lambda }_{s}^{(i)}$ quantifies the vibronic coupling for the *i*-th electronic state. Propagating the nuclear wave packets and using Eq. (8) and (9), the intensity-correlated QFRS signal can be found

11 $$\begin{array}{ll}{S}_{{{{\rm{QE}}}}}(\omega ,{\omega }_{i};T)=\,\frac{N(N-1)}{8{\pi }^{2}}| {{{{\mathcal{E}}}}}_{s,\omega }{| }^{6}| {{{{\mathcal{E}}}}}_{i,{\omega }_{i}}{| }^{2}\\ \qquad\qquad\qquad\times\, {\left|\mathop{\sum}\limits_{i\ne j}\mathop{\sum }\limits_{n = 0}^{\infty }{\alpha }_{{e}_{i},{e}_{j}}^{* }{g}_{n,{e}_{j}}(\omega ,T){\rho }_{{e}_{i},{e}_{j}}^{(n)}(T)\right|}^{2}\end{array}$$

where ${g}_{n,{e}_{j}}(\omega ,T)=\int\nolimits_{-\infty }^{\infty }{{{\rm{d}}}}\tau \,{e}^{i(\omega -{\tilde{\omega }}_{{e}_{i},{e}_{j}}-n{v}_{h})\tau }{e}^{-{D}_{j}({\tau }^{2}+2T\tau )}\tilde{{{\Phi }}}(\tau ,{\omega }_{i})$ and $\tilde{{{\Phi }}}(\tau ,{\omega }_{i})\equiv \frac{1}{2\pi }\int\nolimits_{-\infty }^{\infty }{{\Phi }}(\omega ,{\omega }_{i}){e}^{-i\omega \tau }{{{\rm{d}}}}\omega$, (Supplementary section S3). ${\rho }_{{e}_{i},{e}_{j}}^{(n)}(t)/{\rho }_{{e}_{i},{e}_{j}}(0)=({e}^{-{F}_{j}}{F}_{j}^{n}/n!){e}^{-i({\tilde{\omega }}_{{e}_{i},{e}_{j}}+n{v}_{h})t-{D}_{j}{t}^{2}}$ is the vibronic coherence with *n* harmonics of the high-frequency vibrations having the frequency *v*_*h*_. *F*_*j*_ quantifies the coupling of excitons to high-frequency vibrations whereas *D*_*j*_ quantifies the decay resulting from the coupling to low-energy modes, i.e., solvent and low-frequency vibrations coupled to molecular rotations that form the ro-vibration interaction^64^ (also Supplementary section S3). A superposition yields the electronic coherence, i.e., ${\rho }_{{e}_{i},{e}_{j}}(t)=\mathop{\sum }\nolimits_{n = 0}^{\infty }{\rho }_{{e}_{i},{e}_{j}}^{(n)}(t)$.

Notably, for the timescale $T < 1/\sqrt{{D}_{j}}$, we can find the line-shape function for arbitrary two-photon amplitude, namely, ${g}_{n,{e}_{j}}(\omega ,T)\simeq {{\Phi }}(\omega -{\tilde{\omega }}_{{e}_{i},{e}_{j}}-n{v}_{h}+2i{D}_{j}T,{\omega }_{i})$. Eq. (11) indicates the multiple Raman resonance $\omega -{\omega }_{{{{\rm{pr}}}}}={\tilde{\omega }}_{{e}_{i},{e}_{j}}+n{v}_{h}$ in which the intensity is governed by the Franck–Condon factor quantifying the vibronic interaction. The spectral resolution is ${T}_{s}^{-1}$ that may be greatly enhanced, as dictated by the two-photon amplitude. The Raman signals in Eq. (11) are highly time- and frequency-resolved, revealing the ultrafast coherent dynamics of the electronically excited states.

**Fig. 2:**
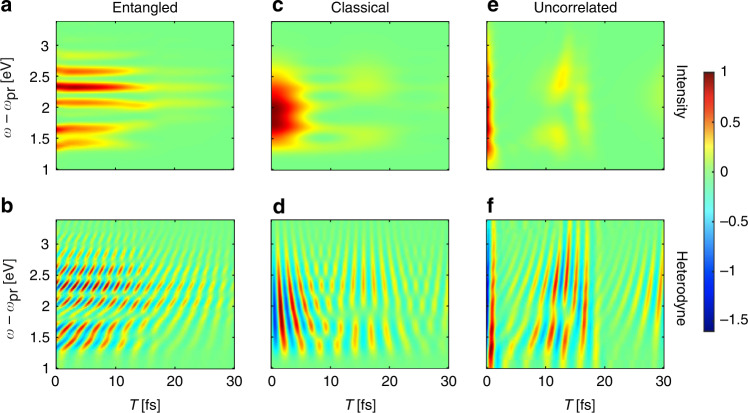
(First column) (**a**) Intensity-correlated QFRS for time-evolving electronic coherence versus the delay *T* between entangled photons and resonant pump pulse; **b** Same as **a** but for heterodyne detection. **c**, **d** Same as **a**, **b** but using classical pulse as probe having bandwidth *σ*_0_ = 0.82eV. **e**, **f** Same as **a**, **b** but using uncorrelated separable state of photons with the bandwidth *σ*_0_ = 0.82eV (${\sigma }_{0}^{-1}=5$fs). *F*_*j*_ and *D*_*j*_ denote the couplings to high-frequency vibrations and other low-energy degrees of freedom; *F*_1_ = 2.2, *F*_2_ = 1.3, ${D}_{1}^{-1/2}=30$fs, ${D}_{2}^{-1/2}=20$fs. Other parameters are taken from 4-oriented amino-4’nitrostilbene^63^, i.e., ${\omega }_{{e}_{1}}=5.3$eV, ${\omega }_{{e}_{2}}=5.7$eV, ${\omega }_{{e}_{3}}=7.1$eV, *v*_*h*_ = 0.26eV and *T*_*s*_ = *T*_*i*_ = 30fs

Figure 2a illustrates the intensity-correlated QFRS, where the electronic coherence ${\rho }_{{e}_{3},{e}_{i}};i=1,2$ is monitored, yielding the Raman resonance corresponding to the two electronically excited states at ${\omega }_{{e}_{1}}=5.3$eV and ${\omega }_{{e}_{2}}=5.7$eV. Using the short pulse, we can readily resolves the vibronic couplings. In particular, the signal contains the Raman resonance $\omega -{\omega }_{{{{\rm{pr}}}}}={\omega }_{{e}_{3},{e}_{1}}+n{v}_{h}\,(n=1,2,3,4)$ and ${\omega }_{{e}_{3},{e}_{2}}+m{v}_{h}\,(m=0,1,2)$, where the peaks at ${\omega }_{{e}_{3},{e}_{1}}+n{v}_{h}$ decay slower and dominate at longer time delay *T*. This is because of the weaker influence from the low-frequency vibrations leading to slower dephasing of electronic coherence. It is worth noting that only a few harmonics of the vibrational modes can be seen from the Raman signal for electronic coherence. This results from the Franck–Condon effect yielding a Poissonian distribution of the vibronic energy seen from *S*_QE_(*ω*, *ω*_*i*_; *T* = 0). Figure 2c illustrates the intensity-correlated QFRS with a classical probe pulse. The broadband pulse has a high temporal resolution, but the individual electronically excited states, as well as their coupling to vibrations are poorly resolved. This has been a long existing bottleneck in ultrafast Raman spectroscopy.

We further examine the Fock state of photons that is fully separable with neither correlation nor entanglement, in Fig. 2e. It shows that the fully separable Fock photon state yields as poor resolutions as the Raman signals with classical probe. This is because the field correlation function is factorized into a product of field intensities.

In general, the highly time-frequency resolved nature of the QFRS is a result from the spectral-resolved idler photons that provides a new parameter space. The idler photons can be resolved independently of the pulse duration which sets the temporal resolution.

It is worth noting that the intensity-correlated QFRS may smear out the phase information of the excited states, as seen from the spectral-resolved condition ${T}_{s}^{-1} \,<\, | {\omega }_{e,e^{\prime} }|$ so that $| {\rho }_{e,e^{\prime} }{| }^{2}$ dominates the signal. A new spectroscopic signal is therefore essential to be exploited, as will be entailed next.

### Femtosecond spectroscopy with entangled light: Heterodyne-detected QFRS

We let the *s*-arm photons serve as a local oscillator interfering with the emitted photons, so that the interference can be recorded in the detectors. This may be achieved by a beam splitter which directs a portion of the photons in *s* arm to propagate freely and subsequently mix with the emission. This is in a fashion of Mach-Zehnder (MZ) interferometer, as depicted in Fig. 1b. The field directed into detector 1 carries the superposition state $\left|e\right\rangle +{e}^{i\phi }\left|e^{\prime} \right\rangle$ assuming 50/50 beam splitters. The joint detection of photons with anti-Stokes shift yields the measurement $\propto {{{\rm{Re}}}}({e}^{-i\phi }{\rho }_{e,e^{\prime} })$. In such a spirit, we find the heterodyne-detected signal explicitly through the transmission of the probe (Supplementary section S2)

12 $${S}_{{{{\rm{HD}}}}}(\omega ,{\omega }_{i};T)=2{{{\rm{Im}}}}\left[\int\nolimits_{-\infty }^{\infty }dt{e}^{i\omega (t-T)}\langle {E}_{i}^{{\dagger} }({\omega }_{i}){E}_{s}^{{\dagger} }(\bar{\omega })\times {E}_{s}(t){E}_{i}({\omega }_{i})\rangle \right]$$

which yields

13 $${S}_{{{{\rm{HD}}}}}(\omega ,{\omega }_{i};T)=N{{{\mathcal{C}}}}{{{\rm{Im}}}}\left[\mathop{\sum}\limits_{e^{\prime} }{\alpha }_{e,e^{\prime} }^{* }{{{\Phi }}}^{* }(\bar{\omega },{\omega }_{i}){f}_{e,e^{\prime} }(T)\right]$$

using Eq. (4), with the same definition of Raman line-shape function and electronic polarizability as in Eq. (8) and (9). ${{{\mathcal{C}}}}=| {{{{\mathcal{E}}}}}_{s,\omega }{| }^{2}| {{{{\mathcal{E}}}}}_{i,{\omega }_{i}}{| }^{2}/\pi$. For the molecules in condensed phases, we incorporate into the signal the nuclear dynamics interacting with electronically excited states. The heterodyne-detected QFRS reads

14 $$\begin{array}{ll}{S}_{{{{\rm{HD}}}}}(\omega ,{\omega }_{i};T)=\,2\pi N{{{\mathcal{C}}}}\mathop{\sum}\limits_{e\ne e^{\prime} }\mathop{\sum }\limits_{n=0}^{\infty }{{{\rm{Im}}}}\left[{\alpha }_{e^{\prime} ,e}^{* }{\rho }_{e^{\prime} ,e}^{(n)}(T)\right.\\ \qquad\qquad\qquad\times\, \left.{{{\Phi }}}^{* }(\bar{\omega },{\omega }_{i}){g}_{n,e}(\omega ,T)\right]\end{array}$$

where *g*_*n*,*e*_(*ω*, *T*) is of the identical definition as in Eq. (11). From Eq. (14), *S*_HD_(*ω*, *ω*_*i*_; *T*) visualizes the full transient coherence dynamics including phase and intensity, via the amplitude ${{\Phi }}(\bar{\omega },{\omega }_{i})$ whose phase varies with the optical path.

**Fig. 3:**
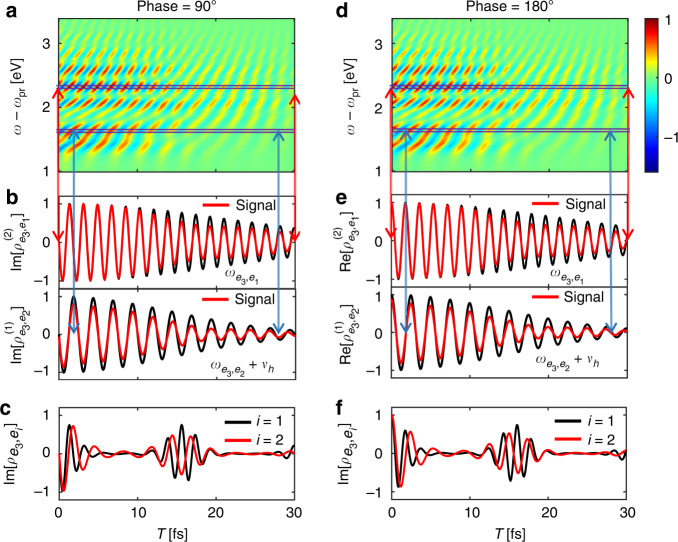
Heterodyne-detected QFRS and dynamics of electronic coherence. (Left column) (**a**) Heterodyne-detected QFRS with the phase associated with ${{\Phi }}(\bar{\omega },{\omega }_{i})$ being − *π*/2 (a certain phase of local oscillator). (b, up) Red line is for the 1D slice of (**a**) at $\omega -{\omega }_{{{{\rm{pr}}}}}={\omega }_{{e}_{3},{e}_{1}}+2{v}_{h}=2.32$eV; Black line is for the imaginary part of *n* = 2 harmonics of the electronic coherence ${\rho }_{{e}_{3},{e}_{1}}$, i.e., ${{{\rm{Im}}}}[{\rho }_{{e}_{3},{e}_{1}}^{(2)}]$. **b** (down) Same as **b** (up) but for $\omega -{\omega }_{{{{\rm{pr}}}}}={\omega }_{{e}_{3},{e}_{2}}+{v}_{h}=1.66$eV and imaginary part of *n* = 1 harmonics of the electronic coherence ${\rho }_{{e}_{3},{e}_{2}}$. **c** Imaginary part of time-evolving electronic coherence, i.e., ${{{\rm{Im}}}}[{\rho }_{{e}_{3},{e}_{1}}]$ (black) and ${{{\rm{Im}}}}[{\rho }_{{e}_{3},{e}_{2}}]$ (red). (Right column) (**d**) Same as (**a**) but for the phase associated with ${{\Phi }}(\bar{\omega },{\omega }_{i})$ being − *π*. **e** Same as (**b**) but for 1D slices of (**d**) (red lines) and ${{{\rm{Re}}}}[{\rho }_{{e}_{3},{e}_{1}}^{(2)}],\,{{{\rm{Re}}}}[{\rho }_{{e}_{3},{e}_{2}}^{(1)}]$ (black lines). **f** Same as (**c**) but for ${{{\rm{Re}}}}[{\rho }_{{e}_{3},{e}_{1}}]$ and ${{{\rm{Re}}}}[{\rho }_{{e}_{3},{e}_{2}}]$. Parameters are the same as Fig. 2

To track the fast coherence dynamics in further, we plot the heterodyne-detected QFRS in Fig. 2b, which unambiguously reveals the oscillations. The heterodyne signal predicts the same Raman resonance as the signal in Fig. 2a. The advantage of photon entanglement is shown by comparing Fig. 2b, d, where Fig. 2d produces a much worse spectral resolution with a classical broadband probe, despite of being temporally resolved. This is extensively supported from the comparison to the heterodyne signal using Fock state of photons, as depicted in Fig. 2e where neither correlation nor entanglement has been involved. Figure 3 shows the heterodyne-detected QFRS with different phases of the local oscillator field that leads to a global phase in ${{\Phi }}(\bar{\omega },{\omega }_{i})$. It can be seen from Fig. 3a, d the fast oscillations that selectively track the real-time dynamics of the coherence phase (imaginary and real parts), given the high spectral resolution revealing the vibronic states. As time evolves, Fig. 3b, e illustrate the Fourier components of the vibronic coherence (imaginary and real parts) resolved from the oscillations with various frequencies, e.g., ${\omega }_{{e}_{3},{e}_{1}}+2{v}_{h}$ and ${\omega }_{{e}_{3},{e}_{2}}+{v}_{h}$ as a result of the vibronic coupling. This leads to the global beating feature of the vibronic coherence, depicted in Fig. 3c and f revealing the phase information.

## Discussion

Since the experiments using electronically ground states are easier and have been accomplished, we will focus on the feasibility of Quantum FAST CARS. The vibrational coherence is scattered off by the *s*-arm photons entangled with the idler photons having a delay from the Stokes pulse. The arrangement of laser beams has to be managed to satisfy the mode matching (MM) condition for the probe beam with respect to the pump-Stokes beams, using a set of MM lens. Dramatically different from the FAST CARS, the probe pulse using entangled twin photons can be broadband, rather than the narrow-band one. Typically, the time duration of fast probe is taken to be ${\sigma }_{0}^{-1}=35$fs and the delay controlled by the crystal length is *T*_*s*_ = 1ps yielding the Raman signal with frequency resolution of 33 cm^−1^ sufficient for a variety of organic molecules. This allows to monitor the fast-evolving vibrational coherence, provided the same spectral resolution as the FAST CARS. Given the time delay ~1 ps between Stokes and probe pulses, the intramolecular vibrational energy relaxation having a typical timescale longer than a few 10s picoseconds is not significant.

We employ the methane (CH_4_) which is a ubiquitous molecule with well characterized spectral lines. The Raman-active transition A_1_ at 2914 cm^−1^ of methane, for instance, can be probed with focusing the femtosecond pump and Stokes pulses followed by *s*-arm photons. The *s*-arm photon pulse intensity is of 10^−9^ magnitude lower than the classical pulse. In a typical CARS scheme the electronic transition dipole is ~1 debye with pump detuning on the order of 10^5^ cm^−1^, the quantum light signal is only ~10^−1^/s. Nevertheless, approaching resonance by reducing detuning with 2 orders, may give an extra 4 orders enhancement of the signal without laser damage. One can then expect the quantum FAST CARS signal at a level of 10^3^/s, which is sufficient for the detection. We note here that the quantum FAST CARS scheme possesses an inherent advantage due to the multi-photon coincidence counting. This plays an essential role in increasing the signal-to-noise ratio for quantum FAST CARS.

In conclusion, we developed a new time- and frequency-resolved Raman spectroscopy using entangled photons and interferometry, as a quantum extension of FAST CARS. Substantially, QFRS was further proposed to probe the electronic coherence. In contrast to existing paradigms^25,31,34^, QFRS is sensitive to the electronic coherence only and is population-free, making it uniquely suitable for monitoring the electronically excited state dynamics during a short timescale. We developed a microscopic theory for the signals involving the intensity- and heterodyne-detected modes. These demonstrated that QFRS can measure the fluctuating energy gap between the vibronic states with a high spectral resolution. The rapid oscillation and decay of electronic coherence can be fully visible in the time-resolved spectrum. Such temporal- and spectral-resolved nature is not attainable in conventional Raman spectra. The QFRS, as a new coherent Raman technique, offers new perspective for the ultrafast dynamics in photophysical systems including low-dimensional semiconductor materials, exciton polaritons and nano-plasmonics.

## Materials and methods

### Generic formalism for QFRS signal

Proceeding via the procedures in Eqs. (1–7), the most general form of the QFRS signal can be found

15 $$\begin{array}{ll}S(\omega ,{\omega }_{i};T)=\,\frac{| {{{{\mathcal{E}}}}}_{s,\omega }{| }^{4}}{4{\pi }^{2}}\mathop{\sum}\limits_{e^{\prime} ,e^{\prime\prime} }\mathop{\sum}\limits_{j\ne j^{\prime} }{\alpha }_{e^{\prime} ,e}^{(j)}{\alpha }_{e,e^{\prime\prime} }^{(j^{\prime} )}\\ \qquad\qquad\quad\times\, \int\nolimits_{-\infty }^{t}{{{\rm{d}}}}\tau \int\nolimits_{-\infty }^{t}{{{\rm{d}}}}\tau ^{\prime} {\rho }_{e,e^{\prime} }^{(j)}(\tau ){\rho }_{e,e^{\prime\prime} }^{(j^{\prime} ),* }(\tau ^{\prime} ){C}_{4}(\tau ^{\prime} ,\tau )\\ \end{array}$$

where ${C}_{4}(\tau ^{\prime} ,\tau )=\left\langle {{\Phi }}\right|{E}_{s}^{{\dagger} }(\tau ^{\prime} ){E}_{s}(\tau ){E}_{i}^{{\dagger} }({\omega }_{i}){E}_{i}({\omega }_{i})\left|{{\Phi }}\right\rangle {e}^{i\omega (\tau -\tau ^{\prime} )}$ is the 4-point field correlation function. It reads very differently for the light field with various photon statistics, as shown in Eq. S7, S14, S19 and S21. In particular, for entangled twin photons generated by SPDC process, one has

16 $${C}_{4}(\tau ^{\prime} ,\tau )=| {{{{\mathcal{E}}}}}_{s,\omega }{| }^{4}{e}^{i\omega (\tau -\tau ^{\prime} )}{\tilde{{{\Phi }}}}^{* }(\tau ^{\prime} -T,{\omega }_{i})\tilde{{{\Phi }}}(\tau -T,{\omega }_{i})$$

where $$\tilde{{{\Phi }}}(\tau -T,{\omega }_{i})={(2\pi )}^{-1}\int {e}^{-i\omega (\tau -T)}{{\Phi }}(\omega ,{\omega }_{i}){{{\rm{d}}}}\omega$$

For psuedo-thermal light, the density matrix has the form ${\rho }_{{{{\rm{pht}}}}}={\sum }_{v}{\sum }_{{v}_{i}}| {{\Phi }}(v,{v}_{i}){| }^{2}\left|{1}_{s,v};{1}_{i,{v}_{i}}\right\rangle \left\langle {1}_{s,v};{1}_{i,{v}_{i}}\right|$ which is classically correlated. The field correlation then reads

17 $$\begin{array}{ll}{C}_{4}(\tau ^{\prime} ,\tau )=\,{{{\rm{Tr}}}}\left[{\rho }_{{{{\rm{pht}}}}}{E}_{s}^{{\dagger} }(\tau ^{\prime} ){E}_{s}(\tau ){E}_{i}^{{\dagger} }({\omega }_{i}){E}_{i}({\omega }_{i})\right]{e}^{-i\omega (\tau ^{\prime} -\tau )}\\ \qquad\qquad=\,\frac{| {{{{\mathcal{E}}}}}_{s,\omega }{| }^{2}| {{{{\mathcal{E}}}}}_{i,{\omega }_{i}}{| }^{2}}{4{\pi }^{2}}\mathop{\sum}\limits_{{\omega }_{2}}{e}^{i(\omega -{\omega }_{2})(\tau -\tau ^{\prime} )}| {{\Phi }}({\omega }_{2},{\omega }_{i}){| }^{2}\end{array}$$

For the photons at Fock state where no correlation survives between the s and i beams, one has Φ(*ω*_*s*_, *ω*_*i*_) = Φ_*s*_(*ω*_*s*_ − *ω*_0_)Φ_*i*_(*ω*_*i*_ − *ω*_0_). The field correlation thus reads

18 $$\begin{array}{ll}{C}_{4}(\tau ^{\prime} ,\tau )=\,| {{{{\mathcal{E}}}}}_{s,\omega }{| }^{4}{e}^{i(\omega -{\omega }_{0})(\tau -\tau ^{\prime} )}{\tilde{{{\Phi }}}}_{s}(\tau -T){\tilde{{{\Phi }}}}_{s}^{* }(\tau ^{\prime} -T)\\ \qquad\qquad\times\, | {{{\Phi }}}_{i}({\omega }_{i}-{\omega }_{0}){| }^{2}\end{array}$$

where ${\tilde{{{\Phi }}}}_{s}(\tau -T)={(2\pi )}^{-1}\int {e}^{-iv(\tau -T)}{{{\Phi }}}_{s}(v){{{\rm{d}}}}v$. Substituting field correlations with different statistics into Eq. (15) we will obtain the Raman signal in the three columns in Fig. 2.

### Microscopic model for QFRS

The Fröhlich–Holstein model (10) for describing molecules in realistic materials involving inhomogeneous dephasing and ro-vibrational coupling undergoes a nuclear wave packet dynamics, which gives the electronic coherence

19 $${\rho }_{e,{e}_{j}}(t)/{\rho }_{e,{e}_{j}}(0)={{{\rm{Tr}}}}\left[{e}^{i{H}_{{{{\rm{vib}}}}}^{j}(p,q)t}{e}^{-i{H}_{{{{\rm{vib}}}}}^{e}(p,q)t}{\rho }_{{{{\rm{vib}}}}}^{e}\right]$$

with ${\rho }_{{{{\rm{vib}}}}}^{e}={Z}^{-1}{\prod }_{s}{e}^{-{v}_{s}{\tilde{b}}_{s}^{{\dagger} }{\tilde{b}}_{s}/T},\,{\tilde{b}}_{s}={b}_{s}-{\lambda }_{s}^{(e)}$, where ${H}_{{{{\rm{vib}}}}}^{j}(p,q)$ is given by Eq. S30b in SI. We then define the energy-gap Hamiltonian ${H}_{-}={H}_{{{{\rm{vib}}}}}^{j}-{H}_{{{{\rm{vib}}}}}^{e}$ where ${H}_{{{{\rm{vib}}}}}^{e}={\sum }_{s}{v}_{s}({\tilde{b}}_{s}^{{\dagger} }{\tilde{b}}_{s}-{\lambda }_{s}^{(e),2})$ and ${\omega }_{e,g} \,>\, {\omega }_{{e}_{j},g}$ as we are interested in anti-Stokes emission. *H*_−_ describes the time-dependent fluctuation as a result of the interaction with vibrations. This essentially results in the inhomogeneous line broadening and statistics of the excited- state transitions. We recast Eq. (19) into

20 $${\rho }_{e,{e}_{j}}(t)/{\rho }_{e,{e}_{j}}(0)={e}^{-i({\omega }_{e,{e}_{j}}+{\bar{\omega }}_{e,{e}_{j}})t}{\left\langle \hat{{{{\mathcal{T}}}}}{e}^{i\int\nolimits_{0}^{t}{H}_{-}(t^{\prime} )dt^{\prime} }\right\rangle}_{{{{\rm{vib}}}}}$$

with ${H}_{-}(t^{\prime} )={e}^{-i{H}_{{{{\rm{vib}}}}}^{e}t}{H}_{-}{e}^{i{H}_{{{{\rm{vib}}}}}^{e}t}$. Using the cumulant expansion we find

21a $${\rho }_{e,{e}_{j}}(t)={\rho }_{e,{e}_{j}}(0){e}^{-i{\tilde{\omega }}_{e,{e}_{j}}t-{g}_{j}(t)}$$

21b $$\begin{array}{ll}{g}_{j}(t)=-\mathop{\sum}\limits_{s}{\left({\lambda }_{s}^{(j)}-{\lambda }_{s}^{(e)}\right)}^{2}\left[\coth \frac{{v}_{s}}{2T}(\cos {v}_{s}t-1)-i\sin {v}_{s}t\right]\end{array}$$

where all the details are referred to Eqs. S30–S34 in SI.

The line-shape function *g*_*j*_(*t*) is partitioned into *g*_*j*_(*t*) = *g*_*j*,*l*_(*t*) + *g*_*j*,*h*_(*t*) for low- and high-frequency vibrations that behave very differently. For the low-frequency vibrations, typically of the order of~100 cm^−1^, *v*_*s*_/*T* ≪ 1, *v*_*s*_*t* ≪ 1 yielding $\coth ({v}_{s}/2T)\simeq 2T/{v}_{s},\,\cos {v}_{s}t\simeq 1-({v}_{s}^{2}{t}^{2}/2),\,\sin {v}_{s}t\simeq {v}_{s}t$. Thus *g*_*j*,*l*_(*t*) ≈ *iD*_*j*_*v*_*l*_*t* + *D*_*j*_*Tv*_*l*_*t*^2^ where ${D}_{j}=m{({\lambda }_{l}^{(j)}-{\lambda }_{l}^{(e)})}^{2}$ with *m* being the number of low-frequency modes. For the high-frequency vibrations undergoing a underdamped dynamics, one has to take into account their discrete nature observed in the spectral density. Normally we can assume *v*_*s*_ ≫ *T* so that $\coth ({v}_{s}/2T)\approx 1$. The line-shape function for a single vibrational mode thus reads ${g}_{j,h}(t)\approx -{F}_{j}\left({e}^{-i{v}_{h}t}-1\right)$ where ${F}_{j}={({\lambda }_{h}^{(j)}-{\lambda }_{h}^{(e)})}^{2}$. Therefore the line-shape function has the approximate form

22 $${g}_{j}(t)\approx i{\bar{v}}_{j,l}t+{D}_{j}{t}^{2}-{F}_{j}\left({e}^{-i{v}_{h}t}-1\right)$$

which gives rise to the electronic coherence

23 $$\begin{array}{l}{\rho }_{e,{e}_{j}}(t)=\mathop{\sum }\limits_{n=0}^{\infty }{\rho }_{e,{e}_{j}}(0)\left({e}^{-{F}_{j}}\frac{{F}_{j}^{n}}{n!}\right){e}^{-i({\tilde{\omega }}_{e,{e}_{j}}+n{v}_{h})t-{D}_{j}{t}^{2}}\end{array}$$

Eq. (23) is essential for computing the Raman signal in Eq. (15). All the details are given in section S3 of SI.

## Supplementary information

Supplementary Information
