# Supplementary material for: Entangled photons enabled time-frequency-resolved coherent Raman spectroscopy and applications to electronic coherences at femtosecond scale
Source: Light Sci Appl. 2022 Sep 14;11:274. doi: 10.1038/s41377-022-00953-y (PMC9474554; doi:10.1038/s41377-022-00953-y)
Supplement: Supplementary file 1 — Supplementary Information [file 41377_2022_953_MOESM1_ESM.pdf]

Supplementary Information for "Entangled Photons Enabled Time-Frequency-Resolved Coherent Raman Spectroscopy and Applications to Electronic Coherences at Femtosecond Scale"

Zhedong Zhang,[1,2,3,*] Tao Peng,[3] Xiaoyu Nie,[4] Girish S. Agarwal,[3,5] and Marlan O. Scully[3,6]

[1]*Department of Physics, City University of Hong Kong, Kowloon, Hong Kong SAR, China*
[2]*City University of Hong Kong, Shenzhen Research Institute, Shenzhen, Guangdong 518057, China*
[3]*Institute for Quantum Science and Engineering, Texas A&M University, College Station, Texas 77843, United States*
[4]*School of Physics, Xi'an Jiaotong University, Xi'an, Shaanxi 710049, China*
[5]*Department of Biological and Agricultural Engineering, Texas A&M University, College Station, Texas 77843, United States*
[6]*Baylor University, Waco, Texas 76704, United States*

(Dated: August 4, 2022)

The math details in addition to the conclusions delivered in main text are provided. Meanwhile, some supplementary results on quantum Raman spectroscopy are shown, in support of the main text.

* zzhan26@cityu.edu.hk

I. INTENSITY-CORRELATED RAMAN SIGNAL WITH ENTANGLED TWIN PHOTONS

In the present study, we employ a single-photon probe pulse produced by a type-II parametric down conversion via a beta barium borate (BBO) crystal. The entangled photon pairs are essentially generated when the BBO crystal is pumped by a broadband pulse. The photon pairs are separated by a beam splitter conserving the energy as well as the momentum. They are then directed into two arms in the fashion of the Hanbury-Brown-Twiss interferometer. With the off-resonant condition, the field-molecule interaction reads ($\hbar = 1$)

$$V(t) = \sum_{j=1}^{N}\sum_{b} \alpha_{bg}^{(j)} (|b\rangle\langle g|)_j E_s(t) E_s^\dagger(t) e^{i(\omega_b-\omega_g)t} + \text{h.c.} \tag{S1a}$$

$$\alpha_{bg}^{(j)} = \sum_{k} \mu_{bk}^{(j)} \mu_{kg}^{(j)} \left(\frac{1}{\omega_k - \omega_g - \omega} + \frac{1}{\omega_k - \omega_b + \omega_{\text{pr}}} \right) \tag{S1b}$$

$$E_s(t) = \frac{1}{2\pi} \sum_{s} E_s(v) e^{-iv(t-T)}, \quad E_s(v) = \mathcal{E}_{s,v} a_{s,v} \tag{S1c}$$

where $\mathcal{E}_{s,v}$ has dimension of electric field. When $E_s(t)$ is written in terms of Stokes and anti-Stokes components, it would reduce to Eq.(2) of Ref.[1]. T denotes the delay relative to the initial preparation of coherence. $[a_{s,v}\,,\, a_{s,v'}^\dagger] = \delta_{v,v'}$ and $\alpha_{bg}^{(j)}$ is the so-called Raman polarizability for electronically ground states [1, 2]. $\mu_{\cdots}^{(j)}$ represents the transition dipole of the jth molecule. In what follows, we assume the identical molecules so that the index j can be eliminated, i.e., $\alpha_{bg}^{(j)} = \alpha_{bg}$.

Since we are interested in the AS component in the emission detected in the spectrometer, the interaction in Eq.(S1a) dictates the full dynamics of the joint molecule-field system. Using Heisenberg's equation of motion, we find

$$\dot{a}_{AS,\omega} = -i[a_{AS,\omega}, V(t)] = \frac{1}{2\pi i} \sum_{j=1}^{N}\sum_{b} \alpha_{bg}^{(j),*} \mathcal{E}_{AS,\omega} (|g\rangle\langle b|)_j E_s(t) e^{i\omega(t-T)} e^{-i(\omega_b-\omega_g)t} \tag{S2}$$

which yields

$$a_{AS,\omega} = \frac{1}{2\pi i} \sum_{j=1}^{N}\sum_{b} \alpha_{bg}^{(j),*} \mathcal{E}_{AS,\omega} (|g\rangle\langle b|)_j \int_{-\infty}^{t} \mathrm{d}\tau E_s(\tau) e^{i\omega(\tau-T)} e^{-i(\omega_b-\omega_g)\tau} \tag{S3a}$$

$$\begin{aligned} n_{AS,\omega} = a_{AS,\omega}^\dagger a_{AS,\omega} &= \frac{1}{4\pi^2} \sum_{j,j'=1}^{N} \sum_{b,b'} \alpha_{bg}^{(j),*} \alpha_{b'g}^{(j')} |\mathcal{E}_{AS,\omega}|^2 (|b'\rangle\langle g|)_{j'} (|g\rangle\langle b|)_j \\ &\times \int_{-\infty}^{t} \mathrm{d}\tau \int_{-\infty}^{t} \mathrm{d}\tau' E_s^\dagger(\tau') E_s(\tau) e^{-i\omega(\tau'-\tau)} e^{i(\omega_{b'}-\omega_g)\tau'} e^{-i(\omega_b-\omega_g)\tau}. \end{aligned} \tag{S3b}$$

The frequency-resolved coincidence counting with the idler photons as a reference leads to the signal, i.e., $S(\omega, \omega_i; T) = |\mathcal{E}_{AS,\omega}|^2 |\mathcal{E}_{i,\omega_i}|^2 \langle n_{AS,\omega} n_{i,\omega_i} \rangle$. Inserting Eq.(S3) one has

$$S(\omega, \omega_i; T) = \frac{|\mathcal{E}_{AS,\omega}|^4}{4\pi^2} \sum_{b,b'} \alpha_{b'g} \alpha_{bg}^* \int_{-\infty}^{t} \mathrm{d}\tau \int_{-\infty}^{t} \mathrm{d}\tau' \bigg(\sum_{j} \rho_{bb'}^{(j)} + \sum_{j\neq j'} \rho_{b'g}^{(j'),*} \rho_{bg}^{(j)} \bigg) e^{i(\omega_{b'}-\omega_g)\tau'} e^{-i(\omega_b-\omega_g)\tau} C_4(\tau', \tau) \tag{S4}$$

with the 4-point field correlation function

$$C_4(\tau', \tau) = \langle \Psi | E_s^\dagger(\tau') E_s(\tau) E_i^\dagger(\omega_i) E_i(\omega_i) | \Psi \rangle e^{-i\omega(\tau'-\tau)} \tag{S5}$$

Eq.(S4) and (S5) provide the most general formalism for the Raman signal with quantum light and nonlinear interferometry. It may be elaborated by the loop diagrams in Fig.S1, distinct from the ones for the femtosecond stimulated Raman scattering (FSRS) with entangled photons [3] and the CARS with classical laser pulses [4]. The spectral properties of the quantum optical fields as given by the field correlation function may play a significant role in accessing the unusual spectroscopic properties of Raman signals.

The photon state produced by BBO consists of a vacuum and two-photon state with one photon in the s arm and another one in the i arm. It is described by the wave function

$$|\Psi\rangle = |0\rangle + \sum_{\omega_s}\sum_{\omega_i} \Phi(\omega_s, \omega_i) a_{\omega_s}^\dagger a_{\omega_i}^\dagger |0\rangle, \tag{S6}$$

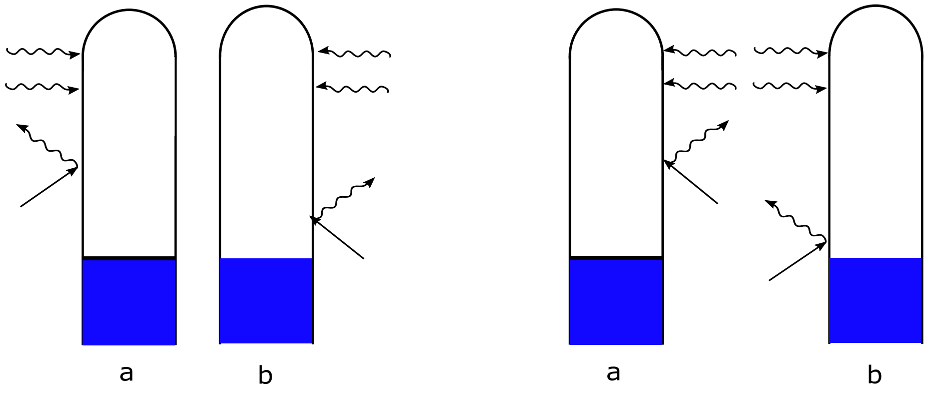

FIG. S1: Loop diagrams for intensity-correlated Raman signal where a and b denote two individual molecules. Solid and wavy arrows represent the probe and emission fields of photons in s arm. The horizontal wavy arrows on the top represent the coincidence counting detection of s and i photons. The Correlation functions corresponding to left and right panels are complex conjugated with each other. The area shaded in blue represents the free propagation before the action of probe field in s arm.

where $a^\dagger_{\omega_s}(a^\dagger_{\omega_i})$ denotes the creation operator of the photons in s (i) arm and the two-photon amplitude follows Eq.(2) in the main text. Inserting Eq.(S6) into (S5) and dropping the terms from vacuum mode and converting the summation into integration eventually we obtain (it will be borne in mind that the summations would be converted to integrals in the last step)

$$C_4(\tau',\tau) = \frac{|\mathcal{E}_{i,\omega_i}|^2}{4\pi^2}\iint d\omega_1' d\omega_2 \mathcal{E}_{s,\omega_1'}\mathcal{E}^*_{s,\omega_2}\Phi^*(\omega_2,\omega_i)\Phi(\omega_1',\omega_i)e^{i\omega_2(\tau'-T)}e^{i\omega_1'(\tau-T)}e^{i\omega(\tau-\tau')}$$

$$\simeq \frac{|\mathcal{E}_{AS,\omega}|^2|\mathcal{E}_{i,\omega_i}|^2}{4\pi^2}\iint d\omega_1' d\omega_2 e^{i(\omega-\omega_1')(\tau-T)}e^{-i(\omega-\omega_2)(\tau'-T)}\Phi^*(\omega_2,\omega_i)\Phi(\omega_1',\omega_i). \tag{S7}$$

The coherent Raman process arises from the cooperative motion of molecular pairs [5, 6], as seen from the coherence term $\sum_{j\neq j'}\rho^{(j'),*}_{b'g}\rho^{(j)}_{bg}$ in Eq.(S4). The product of coherences indicates that the molecules have to be in phase with each other, so as to generate nonzero coherent Raman signal. We substitute Eq.(S7) into (S4) by dropping the spontaneous Raman term $\rho_{bb'}$. The coherent Raman signal is found thereby

$$S(\omega,\omega_i;T) = \frac{|\mathcal{E}_{AS,\omega}|^6|\mathcal{E}_{i,\omega_i}|^2}{16\pi^4}\sum_{j\neq j'}\sum_{b,b'}\alpha_{b'g}\alpha^*_{bg}\int_{-\infty}^t d\tau\int_{-\infty}^t d\tau'\iint d\omega_1 d\omega_2\ \rho^{(j'),*}_{b'g}\rho^{(j)}_{bg}e^{i(\omega_{b'}-\omega_g)\tau'}e^{-i(\omega_b-\omega_g)\tau}$$

$$\times\, e^{i(\omega-\omega_1)(\tau-T)}e^{-i(\omega-\omega_2)(\tau'-T)}\Phi(\omega_1,\omega_i)\Phi^*(\omega_2,\omega_i) \tag{S8}$$

$$= \frac{N(N-1)}{16\pi^4}|\mathcal{E}_{AS,\omega}|^6|\mathcal{E}_{i,\omega_i}|^2\left|\sum_b \alpha^*_{bg}f_{bg}(T)\right|^2$$

with the Raman line-shape function defined as

$$f_{bg}(T) = \int_{-\infty}^t d\tau\int_{-\infty}^\infty d\omega'\rho_{bg}(\tau)e^{i(\omega-\omega')(\tau-T)}\Phi(\omega',\omega_i) \tag{S9a}$$

$$\rho_{bg}(\tau) = \rho_{bg}e^{-[i(\omega_b-\omega_g)+\mathrm{sgn}(\tau)\gamma_{bg}]\tau}\ \text{for vibration coherence.} \tag{S9b}$$

Eq.(S8) and (S9) reveal the coherent Raman signal monitoring the quantum coherence. The math manipulations can be readily generalized to electronic coherence having the dynamics more complicated than the pure exponential decay of the vibrational coherence [4, 7]. This will be elaborated later where the Raman signal shares the formalism with Eq.(S8) for the electronic coherence involved in the Raman line-shape function for electronically excited states.

Substituting the vibrational coherence in Eq.(S9b) one can calculate the Raman line-shape function

$$f_{bg}(T) = \rho_{bg}(T)\int_{-\infty}^\infty d\omega'\frac{e^{[i(\omega-\omega'-\omega_{bg})+\gamma_{bg}](t-T)}}{i(\omega-\omega'-\omega_{bg})+\gamma_{bg}}\Phi(\omega',\omega_i)$$

$$= 2\pi\theta(t-T)\rho_{bg}(T)\Phi(\omega-\omega_{bg}-i\gamma_{bg},\omega_i) \tag{S10}$$

where the integral has been completed via the contour integration in complex domain. Substituting Eq. (S10) into Eq. (S8) we obtain the Quantum FAST CARS signal

$$S_{\rm QV}(\omega,\omega_i;T)=\frac{N(N-1)}{4\pi^2}|\mathcal{E}_{AS,\omega}|^6|\mathcal{E}_{i,\omega_i}|^2\left|\sum_b \alpha^*_{bg}\rho_{bg}(T)\Phi(\omega-\omega_{bg}-i\gamma_{bg},\omega_i)\right|^2 \tag{S11}$$

as given in Eq.(3) in the main text, where $\gamma_{bg}<T_s^{-1}$. Eq.(S11) indicates a spectral resolution T_s^{-1} when considering the optimal photon entanglement given $T_i=T_s$, as entailed in the main text. The two-photon amplitude thereby reads

$$\Phi(\omega_s,\omega_i)=A_0 e^{-\frac{(\omega_s+\omega_i-\omega_0)^2}{2\sigma_0^2}}\,{\rm sinc}\left[(\omega_s+\omega_i-\omega_0)\frac{T_s}{2}\right] \tag{S12}$$

assuming a Gaussian profile for the pump pulse.

To see the photon entanglement closely, we essentially calculate the entanglement entropy for photon pairs, according to $E(\Phi)=-\sum_k\lambda_k^2\ln(\lambda_k^2)$. λ_k denotes the normalized set of the eigenvalues in Schmidt decomposition of the two-photon amplitude such that $\sum_k\lambda_k^2=1$ and

$$\Phi(\omega_s,\omega_i)=\sqrt{B}\sum_{k=1}^{\infty}\lambda_k\psi_k^*(\omega_s)\phi_k^*(\omega_i) \tag{S13}$$

where $\{\psi_k\}$ and $\{\phi_k\}$ form orthonormal basis, known as the Schmidt modes with normalization constant B [8, 9]. Fig.S2(a)-(e) show the entanglement entropy of the photon wave function based on Eq.(S13). The optimal quantum entanglement between s and idler photons is observed when they arrive simultaneously, regardless of the pump pulse bandwidth. By varying the bandwidth of the pump pulse, one can see that with a time delay the photon pair is allowed to be entangled considerably when using a broad-band SPDC pump field, while the photon entanglement drastically drops when using a narrow-band SPDC pump field. This can be further understood from the eigenvalue distribution of the two-photon density matrix under the Schmidt decomposition, as depicted in Fig.S2(f). The photon entanglement with flexible time delay may provide a promising advantage of using entangled photons to produce ultrafast time-resolved spectroscopic signals.

For classical probe pulse, the photon wave function is $|\Psi\rangle=|\{\alpha\}\rangle\otimes|\varphi_i\rangle$ where the idler photons are irrelevant in the detection. The Raman signal reads

$$\begin{aligned}S_{\rm CV}(\omega,\omega_i;T)=&\frac{|\mathcal{E}_{AS,\omega}|^4}{4\pi^2}\sum_{b',b}\alpha_{b'g}\alpha^*_{bg}\int_{-\infty}^t d\tau\int_{-\infty}^t d\tau'\Big(\sum_j\rho_{bb'}^{(j)}+\sum_{j\neq j'}\rho_{b'g}^{(j'),*}\rho_{bg}^{(j)}\Big)e^{i(\omega_{b'}-\omega_g)\tau'}e^{-i(\omega_b-\omega_g)\tau}\\&\times\langle\{\alpha\}|E_s^\dagger(\tau')E_s(\tau)|\{\alpha\}\rangle\langle\varphi_i|E_i^\dagger(\omega_i)E_i(\omega_i)|\varphi_i\rangle e^{-i\omega(\tau'-\tau)}\\=&\frac{|\mathcal{E}_{AS,\omega}|^4|\mathcal{E}_{i,\omega_i}|^2 n_{i,\omega_i}}{4\pi^2}\sum_{b',b}\alpha_{b'g}\alpha^*_{bg}\int_{-\infty}^t d\tau\int_{-\infty}^t d\tau'\Big(\sum_j\rho_{bb'}^{(j)}+\sum_{j\neq j'}\rho_{b'g}^{(j'),*}\rho_{bg}^{(j)}\Big)e^{i(\omega_{b'}-\omega_g)\tau'}\\&\times e^{-i(\omega_b-\omega_g)\tau}e^{i\omega(\tau-\tau')}E^*_{\rm pr}(\tau'-T)E_{\rm pr}(\tau-T).\end{aligned} \tag{S14}$$

and $E_s(\tau)|\{\alpha\}\rangle=E_{\rm pr}(\tau-T)|\{\alpha\}\rangle$, $\langle\varphi_i|E_i^\dagger(\omega_i)E_i(\omega_i)|\varphi_i\rangle=|\mathcal{E}_{i,\omega_i}|^2n_{i,\omega_i}$. Using CW field, $E_{\rm pr}(\tau-T)=E_{\rm pr}e^{-iv_{\rm pr}\tau}$ so that

$$S_{\rm CV}(\omega,\omega_i;T)=\frac{|\mathcal{E}_{i,\omega_i}|^2n_{i,\omega_i}}{4\pi^2}\sum_b\frac{|\mathcal{E}_{AS,\omega}|^6|\alpha_{bg}|^2n_{\rm pr}}{(\omega_b-\omega_g+v_{\rm pr}-\omega)^2+\gamma^2}\Big(\sum_j\rho_{bb}^{(j)}+\sum_{j\neq j'}\rho_{bg}^{(j'),*}\rho_{bg}^{(j)}\Big) \tag{S15}$$

keeping the most significant terms where $|E_{\rm pr}|^2=|\mathcal{E}_{AS,\omega}|^2n_{\rm pr}$. Eq.(S15) coincides with the results in Ref.[2], i.e.,

$$S_{\rm CV}(\omega_b-\omega_g+v_{\rm pr},\omega_i;T)={\rm const.}\times\frac{|\mathcal{G}|^2n_{\rm pr}}{\gamma^2}\Big(\sum_j\rho_{bb}^{(j)}+\sum_{j\neq j'}\rho_{gb}^{(j')}\rho_{bg}^{(j)}\Big)\times n_{i,\omega_i} \tag{S16}$$

along with the two-photon resonance condition $\omega_b-\omega_g+v_{\rm pr}-\omega=0$ and $\mathcal{G}=\mathcal{E}^2_{AS,\omega}\alpha_{bg}$. $S_{\rm CV}$ is thus a product of Eq.(S11) in Ref.[2] and idler intensity.

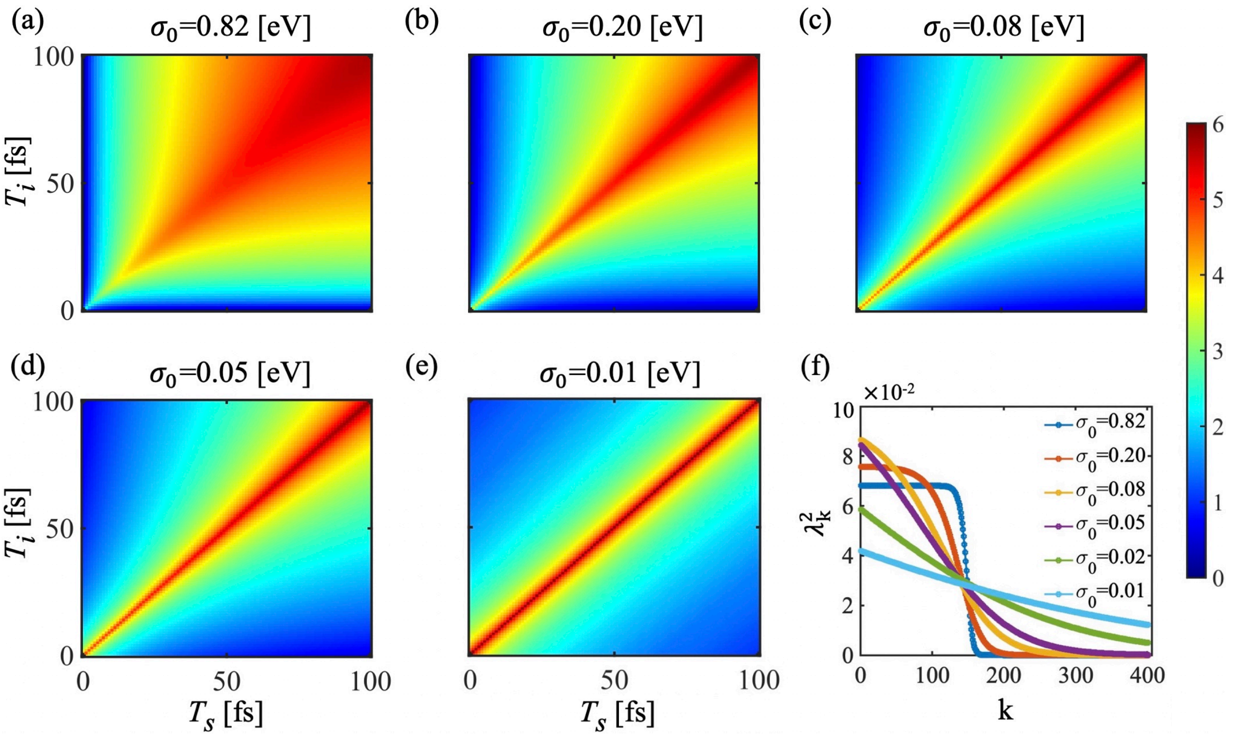

FIG. S2: (a)-(e) Entanglement entropy of twin photons as a function of time delays between s & i photons and the SPDC pump, calculated from Schmidt decomposition; σ_0 denotes the bandwidth of SPDC pump. (f) Spectrum of the eigenvalues λ_k^2 in Schmidt decomposition for the entangled state with the amplitude Eq.(S13) assuming $T_s = T_i = 30$fs.

Using the classical broadband pulse as probe, i.e., $E_{\rm pr}(\tau - T) = E_{\rm pr} e^{-i v_{\rm pr}(\tau - T)} \delta(\tau - T)$, the Raman signal is

$$S_{\rm CV}(\omega, \omega_i; T) = \frac{|\mathcal{E}_{AS,\omega}|^6 |\mathcal{E}_{i,\omega_i}|^2 n_{\rm pr} n_{i,\omega_i}}{4\pi^2} \sum_{b',b} \alpha_{b'g} \alpha^*_{bg} \left[\sum_j \rho^{(j)}_{bb'}(T) + \sum_{j \neq j'} \rho^{(j'),*}_{b'g}(T) \rho^{(j)}_{bg}(T) \right] \tag{S17}$$

which is highly time-resolved but not frequency-resolved.

Next we compare the Raman signal using entangled twin photons with the cases using incoherent light. For the sake of simplicity, we focus on the Quantum FAST CARS, whereas the conclusions will be the same for the excited-state Raman signal. Two incoherent states of light will be of our interest: (i) the psuedo-thermal light and (ii) the photons at Fock state. For (i), the density matrix of photon is

$$\rho_{\rm pht} = \sum_v \sum_{v_i} |\Phi(v, v_i)|^2 |1_{s,v}; 1_{i,v_i}\rangle \langle 1_{s,v}; 1_{i,v_i}| \tag{S18}$$

which involves the classical correlation. The 4-point field correlation function then reads

$$C_4(\tau', \tau) = {\rm Tr}\left[\rho_{\rm pht} E^\dagger_s(\tau') E_s(\tau) E^\dagger_i(\omega_i) E_i(\omega_i) \right] e^{-i\omega(\tau' - \tau)} = \frac{|\mathcal{E}_{s,\omega}|^2 |\mathcal{E}_{i,\omega_i}|^2}{4\pi^2} \sum_{\omega_2} e^{i(\omega - \omega_2)(\tau - \tau')} |\Phi(\omega_2, \omega_i)|^2 \tag{S19}$$

which loses the phase relation between different modes, compared to Eq.(S7). Inserting Eq.(S19) into Eq.(S4), some algebra finds

$$\begin{aligned} S_{\rm cor}(\omega, \omega_i; T) &= \frac{|\mathcal{E}_{s,\omega}|^6 |\mathcal{E}_{i,\omega_i}|^2}{16\pi^4} \sum_{a,b=1}^{N} \sum_{b_1,b_2} \alpha^{(a),*}_{b_1,g} \alpha^{(b)}_{b_2,g} \int_{-\infty}^{t} {\rm d}\tau \int_{-\infty}^{t} {\rm d}\tau' \int_{-\infty}^{\infty} {\rm d}\omega' \rho^{(a)}_{b_1,g}(\tau) \rho^{(b),*}_{b_2,g}(\tau') e^{i(\omega - \omega')(\tau - \tau')} \left|\Phi(\omega', \omega_i)\right|^2 \\ &= \frac{N(N-1)}{4\pi^3} |\mathcal{E}_{s,\omega}|^6 |\mathcal{E}_{i,\omega_i}|^2 {\rm Im} \sum_{b_1,b_2} \alpha^*_{b_1,g} \alpha_{b_2,g} \rho_{b_1,g} \rho^*_{b_2,g} \frac{|\Phi(\omega - \omega_{b_1,g} - i\gamma_{b_1,g}, \omega_i)|^2}{\omega_{b_1,b_2} - i(\gamma_{b_1,g} + \gamma_{b_2,g})}. \end{aligned} \tag{S20}$$

Eq.(S20) shows that the high spectral resolution T_s^{-1} can be achieved by the classically correlated photons but the temporal resolution is no longer there, as seen from the no T dependence in the field correlation function in Eq.(S19).

For (ii) the Fock state, we have $\Phi(\omega_s,\omega_i)=\Phi_s(\omega_s-\omega_0)\Phi_i(\omega_i-\omega_0)$ where the single-photon amplitude $\Phi_k(\omega_k)\propto e^{-\omega_k^2/2\sigma_0^2}$ has the identical time duration as the pump pulse in Eq.(S12). Thus the signal can be obtained

$$S_{\rm cl}(\omega,\omega_i;T)=\frac{N(N-1)}{4\pi^2}|\mathcal{E}_{s,\omega}|^6|\mathcal{E}_{i,\omega_i}|^2\left|\sum_b\alpha_{bg}^*\rho_{bg}(T)\Phi_s(\omega-\omega_0-\omega_{bg}-i\gamma_{bg})\Phi_i(\omega_i-\omega_0)\right|^2 \tag{S21}$$

which in some sense, may emulate the effect of classical pulses at the single-photon level, as will be manifested later in the excited-state Raman signal. As shown in Eq.(S21), the idler dependence is factorized so that the photons in idler arm are irrelevant.

Nevertheless, it is worth noting that the Raman signal with entangled photons can provide the information about the envelop dynamics of the coherence $\rho_{bg}(T)$ whereas the phase information such as oscillation feature has been smeared out. To see this closely, we recast Eq.(S11) into

$$\begin{aligned}S_{\rm QV}(\omega,\omega_i;T)=&\frac{N(N-1)}{4\pi^2}|\mathcal{E}_{s,\omega}|^6|\mathcal{E}_{i,\omega_i}|^2\Bigg[\sum_b|\alpha_{bg}|^2\,|\rho_{bg}(T)|^2\,|\Phi\left(\omega-\omega_{bg}-i\gamma_{bg},\omega_i\right)|^2\\&+\sum_{b_1\neq b_2}\alpha_{b_1,g}^*\alpha_{b_2,g}\rho_{b_1,g}(T)\rho_{b_2,g}^*(T)\Phi\left(\omega-\omega_{b_1,g}-i\gamma_{b_1,g},\omega_i\right)\Phi^*\left(\omega-\omega_{b_2,g}-i\gamma_{b_2,g},\omega_i\right)\Bigg]\\ \simeq&\frac{N(N-1)}{4\pi^2}|\mathcal{E}_{s,\omega}|^6|\mathcal{E}_{i,\omega_i}|^2\sum_b|\alpha_{bg}|^2\,|\rho_{bg}(T)|^2\,|\Phi\left(\omega-\omega_{bg}-i\gamma_{bg},\omega_i\right)|^2\end{aligned} \tag{S22}$$

where the 2nd step takes the rational once $T_s^{-1}<|\omega_{b_1,b_2}|$ acquired by the enhanced spectral resolution using photon entanglement that results in little overlap between the amplitudes Φ and Φ^*. Eq.(S22) indicates that the phase information associated with the electronic coherence cannot be obtained as desired, due to $|\rho_{bg}(T)|^2$. The same conclusion can be made for the excited-state Raman signal using entangled photons, and this will be a crucial scar for electronic coherence. A new spectroscopic signal is therefore essential to be explored, as will be entailed in next section.

II. HETERODYNE-DETECTED QFRS

To resolve the phase information of the emission from molecules, a local oscillator has to be involved. Analogous to the heterodyne signal with classical pulses, the incident photons in s arm serves as a local oscillator interfering with the emitted photons, so that the interference can be recorded in the detectors. This may be achieved by a beam splitter which directs a portion of the photons in s arm to propagate freely and subsequently mix with the emission. This is in a fashion of Mach-Zehnder (MZ) interferometer, as depicted in Fig.1(c) in the main text, where the MZ scheme generates entangled states in s arm.

To get a closer glance, we note the first beam splitter (BS) gives the operator $(\sqrt{u}a_{s,1}+\sqrt{1-u}a_{s,2})\otimes|e\rangle$ prior to the interaction with the molecules, where the subscripts 1 and 2 correspond to the pathways denoted in Fig.1(c). After the scattering by molecules, the joint state of s photons and molecule becomes $\sqrt{u}a_{s,1}\otimes|e\rangle+\sqrt{1-u}a_{s,2}\otimes|e'\rangle$. The photons in the two pathways may interfere with each other via the second BS which leads to

$$\begin{pmatrix}a_{s,1}\\a_{s,2}\end{pmatrix}=\begin{pmatrix}\sqrt{v}&\sqrt{1-v}\\-\sqrt{1-v}e^{i\phi}&\sqrt{v}e^{i\phi}\end{pmatrix}\begin{pmatrix}b_{s,1}\\b_{s,2}\end{pmatrix} \tag{S23}$$

where the π phase in pathways 2 is due to the half-wave loss of BS-induced reflection. Detecting the photons along the pathway 2 yields $b_{s,2}\otimes\left[\sqrt{u(1-v)}|e\rangle+\sqrt{(1-u)v}e^{i\phi}|e'\rangle\right]$. Thus the quantity related to the pathway interference of photons measured in the detector reads $Q=\sqrt{u(1-v)(1-u)v}\;b_{s,2}^\dagger b_{s,2}\otimes\left(e^{-i\phi}|e\rangle\langle e'|+e^{i\phi}|e'\rangle\langle e|\right)$, which subsequently results in the joint detection of photons in two arms $G=\langle Qa_i^\dagger a_i\rangle$ giving

$$G=2\sqrt{u(1-v)(1-u)v}\;\langle N_{s,2}N_i\rangle{\rm Re}\left(e^{-i\phi}\rho_{e,e'}\right) \tag{S24}$$

with $N_{s,2}=b_{s,2}^\dagger b_{s,2},\;N_i=a_i^\dagger a_i$.

In the spirit above, we write down the heterodyne-detected signal explicitly

$$S_{\rm QEHD}(\omega,\omega_i;T)=2{\rm Im}\left[\int_{-\infty}^{\infty}{\rm d}te^{i\omega(t-T)}\langle E_i^\dagger(\omega_i)E_s^\dagger(\bar{\omega})V^{(-)}E_i(\omega_i)\rangle_{\rho(t)}\right] \tag{S25}$$

where $E_s^\dagger(\bar{\omega})$ is the positive frequency part of the Fourier component of the local oscillator and $V=\sum_e\mu_{eg}|e\rangle\langle g|+\sum_{e,f}\mu_{fe}|f\rangle\langle e|+\text{h.c.}$ denotes the dipole operator. Expanding the signal with respect to molecule-field coupling, one finds

$$S_{\rm QEHD}(\omega,\omega_i;T)=2\sum_{a=1}^{N}{\rm Re}\left[\int_{-\infty}^{\infty}{\rm d}t\int_{-\infty}^{t}{\rm d}\tau e^{i\omega(t-T)}F_a(t-\tau,\tau)C(\tau)\right] \tag{S26}$$

with the Green's functions for molecules and field

$$\begin{aligned}F_a(t-\tau,\tau)&=\sum_{e_1}\sum_{f}\mu_{e_1,f}^{(a)}\mu_{f,e}^{(a)}\;e^{-(i\omega_{f,e_1}^{(a)}+\gamma_{f,e_1})(t-\tau)}\rho_{e,e_1}^{(a)}(\tau)\\ C(\tau)&=\langle\Psi|E_i^\dagger(\omega_i)E_i(\omega_i)E_s^\dagger(\bar{\omega})E_s(\tau)|\Psi\rangle=\frac{|\mathcal{E}_{s,\omega}|^2|\mathcal{E}_{i,\omega_i}|^2}{2\pi}\int_{-\infty}^{\infty}{\rm d}\omega'e^{-i\omega'(\tau-T)}\Phi^*(\bar{\omega},\omega_i)\Phi(\omega',\omega_i)\end{aligned} \tag{S27}$$

in which the superscript a labels the individual molecule in the sample. Inserting Eq.(S27) into Eq.(S26) we proceed with the heterodyne signal

$$\begin{aligned}S_{\rm QEHD}(\omega,\omega_i;T)=&\frac{|\mathcal{E}_{s,\omega}|^2|\mathcal{E}_{i,\omega_i}|^2}{2\pi}\sum_{a=1}^{N}\sum_{e_1}\sum_{f}\int_{-\infty}^{\infty}{\rm d}t\int_{-\infty}^{t}{\rm d}\tau\int_{-\infty}^{\infty}{\rm d}\omega'\mu_{e_1,f}^{(a)}\mu_{f,e}^{(a)}\;e^{i\omega(t-T)}\\ &\times e^{-(i\omega_{f,e_1}^{(a)}+\gamma_{f,e_1})(t-\tau)}e^{-i\omega'(\tau-T)}\rho_{e,e_1}^{(a)}(\tau)\Phi^*(\bar{\omega},\omega_i)\Phi(\omega',\omega_i)+\text{c.c.}\\ =&\frac{N}{\pi}|\mathcal{E}_{s,\omega}|^2|\mathcal{E}_{i,\omega_i}|^2{\rm Im}\Big[\sum_{e_1}\alpha_{e,e_1}^*\Phi^*(\bar{\omega},\omega_i)f_{e,e_1}(T)\Big]\end{aligned} \tag{S28}$$

where f denotes the highly-excited states and the electronic polarizability is found to be

$$\alpha_{e,e_1}=\sum_f\frac{\mu_{e,f}\mu_{f,e_1}}{\hbar}\left(\frac{1}{\omega_{f,e_1}-\omega}+\frac{1}{\omega_{f,e_1}+\omega}\right) \tag{S29}$$

when the field is far-detuned from the electronic transitions. The electronic polarizability in Eq.(S29) is consistent with that given by Eq.(S1).

III. BROWNIAN OSCILLATOR MODEL FOR NUCLEAR DYNAMICS

The pure exponential decay adopted in vibrational coherence is no longer sufficient for studying the fast-evolving electron dynamics of the excited molecules. The molecules in realistic materials, e.g., embedded in dense medium, often involve inhomogeneous dephasing, in which the electronic coherence may play a significant role. This acquires the knowledge on the ultrafast excited-state dynamics coupled to nuclear motion, responsible for rich effects. To this end, we consider the Fröhlich-Holstein model for molecules

$$H=H_{\rm vib}(p,q)|g\rangle\langle g|+\sum_j H_{\rm vib}^j(p,q)|e_j\rangle\langle e_j| \tag{S30a}$$

$$H_{\rm vib}(p,q)=\sum_s v_s b_s^\dagger b_s,\;\;H_{\rm vib}^j(p,q)=\omega_{e_j,g}+\lambda_s^{(j),2}v_s+\sum_s\Big[v_s b_s^\dagger b_s-\lambda_s^{(j)}v_s(b_s+b_s^\dagger)\Big] \tag{S30b}$$

where b_s and $b_s^\dagger$ are the annihilation and creation operators for the s-th vibration having the frequency v_s. $p_s=(b_s^\dagger-b_s)/i\sqrt{2}$, $q_s=(b_s^\dagger+b_s)/\sqrt{2}$ are the dimensionless momentum and coordinate for the vibrations, respectively.

$\lambda_s^{(j)}$ quantifies the vibronic coupling for the j-th electronic state. The propagation of nuclear wave packets yields the electronic coherence

$$\rho_{e,e_j}(t)/\rho_{e,e_j}(0) = \mathrm{Tr}\left[e^{iH^j_{\mathrm{vib}}(p,q)t}e^{-iH^e_{\mathrm{vib}}(p,q)t}\rho^e_{\mathrm{vib}}\right], \quad \rho^e_{\mathrm{vib}} = Z^{-1}\prod_s e^{-v_s\tilde{b}^\dagger_s\tilde{b}_s/T} \tag{S31}$$

with the displaced operator $\tilde{b}_s = b_s - \lambda_s^{(e)}$. To compute the average above, we introduce the nuclear Hamiltonian associated with individual electronic state such that $H^j_{\mathrm{vib}}(p,q) = \omega_{e_j,g} + \lambda_s^{(j),2}v_s + H^j_{\mathrm{vib}}$ and

$$H^j_{\mathrm{vib}} = \sum_s \left[v_s b^\dagger_s b_s - \lambda_s^{(j)}v_s(b_s + b^\dagger_s)\right] \tag{S32}$$

and the energy-gap Hamiltonian $H_- = H^j_{\mathrm{vib}} - H^e_{\mathrm{vib}}$ where $H^e_{\mathrm{vib}} = \sum_s v_s(\tilde{b}^\dagger_s\tilde{b}_s - \lambda_s^{(e),2})$. Here e and e_j denote different electronically excited states such that $\omega_{e,g} > \omega_{e_j,g}$ as we are interested in anti-Stokes emission mostly. H_- describes the time-dependent fluctuation as a result of the interaction with vibrations. The time-average of the correlation function for these energy gap fluctuations leads to the inhomogeneous line broadening and statistics of the excited-state transitions. Eq.(S31) can be recast into

$$\begin{aligned} \rho_{e,e_j}(t)/\rho_{e,e_j}(0) &= e^{-i(\omega_{e,e_j}+\bar{\omega}_{e,e_j})t}\mathrm{Tr}\left(e^{iH^j_{\mathrm{vib}}t}e^{-iH^e_{\mathrm{vib}}t}\rho^e_{\mathrm{vib}}\right) = e^{-i(\omega_{e,e_j}+\bar{\omega}_{e,e_j})t}\left\langle \hat{\mathcal{T}}e^{i\int_0^t H_-(t')dt'}\right\rangle_{\mathrm{vib},e} \\ H_-(t') &= e^{-iH^e_{\mathrm{vib}}t}H_-e^{iH^e_{\mathrm{vib}}t}. \end{aligned} \tag{S33}$$

Using the cumulant expansion we obtain [6, 10]

$$\begin{aligned} \rho_{e,e_j}(t) &= \rho_{e,e_j}(0)e^{-i(\omega_{e,e_j}+\bar{\omega}_{e,e_j})t}\left[1 + i\int_0^t dt'\langle H_-(t')\rangle - \int_0^t dt''\int_0^{t''} dt'\langle H_-(t'')H_-(t')\rangle + \cdots\right] \\ &= \rho_{e,e_j}(0)e^{-i\tilde{\omega}_{e,e_j}t - g_j(t)} \\ g_j(t) &= \int_0^t dt''\int_0^{t''} dt'\Big(\langle H_-(t'')H_-(t')\rangle - \langle H_-(t'')\rangle\langle H_-(t')\rangle\Big) \\ &= -\sum_s\left(\lambda_s^{(j)} - \lambda_s^{(e)}\right)^2\left[\coth\frac{v_s}{2T}(\cos v_s t - 1) - i\sin v_s t\right]. \end{aligned} \tag{S34}$$

For the molecules in condensed phase, the high-frequency vibrations, typically of the order of $\sim 1000\mathrm{cm}^{-1}$, are mostly underdamped whereas the low-frequency ones, typically of the order of $\sim 100\mathrm{cm}^{-1}$, are densely distributed and undergo an overdamped process. To amount for such drastic difference, the line-shape function $g(t)$ can be properly partitioned into $g_j(t) = g_{j,l}(t) + g_{j,h}(t)$ where l and h denote the low-frequency and high-frequency vibrations, respectively [11].

For the low-frequency vibrations, the spectral density is smooth and $v_s/T \ll 1,\ v_s t \ll 1$, leading to $\coth(v_s/2T) \simeq 2T/v_s,\ \cos v_s t \simeq 1 - (v_s^2t^2/2),\ \sin v_s t \simeq v_s t$, and thus

$$g_{j,l}(t) \simeq iD_j v_l t + D_j T v_l t^2 \tag{S35}$$

where $D_j = m(\lambda_l^{(j)} - \lambda_l^{(e)})^2$ with m being the number of low-frequency modes.

For the high-frequency modes undergoing a underdamped dynamics, sharp peaks can be often characterized in the spectral density so that the discrete nature has to be taken into account in calculating the line-shape function. For the sake of simplicity, we assume a single vibrational mode hereafter and the generalization to multiple modes is straightforward. With $v_h \gg T$ under room temperature we have

$$g_{j,h}(t) = -F_j\left[\coth\frac{v_h}{2T}(\cos v_h t - 1) - i\sin v_h t\right] \simeq -F_j\left(e^{-iv_h t} - 1\right) \tag{S36}$$

where $F_j = (\lambda_h^{(j)} - \lambda_h^{(e)})^2$. Adding Eq.(S35) and (S36) the line-shape function reads $g_j(t) = i\bar{v}_{j,l}t + D_j t^2 - F_j(e^{-iv_h t} - 1)$ which gives rise to the electronic coherence

$$\rho_{e,e_j}(t) = \sum_{n=0}^{\infty}\rho_{e,e_j}(0)\left(e^{-F_j}\frac{F_j^n}{n!}\right)e^{-i(\tilde{\omega}_{e,e_j}+nv_h)t - D_j t^2} \tag{S37}$$

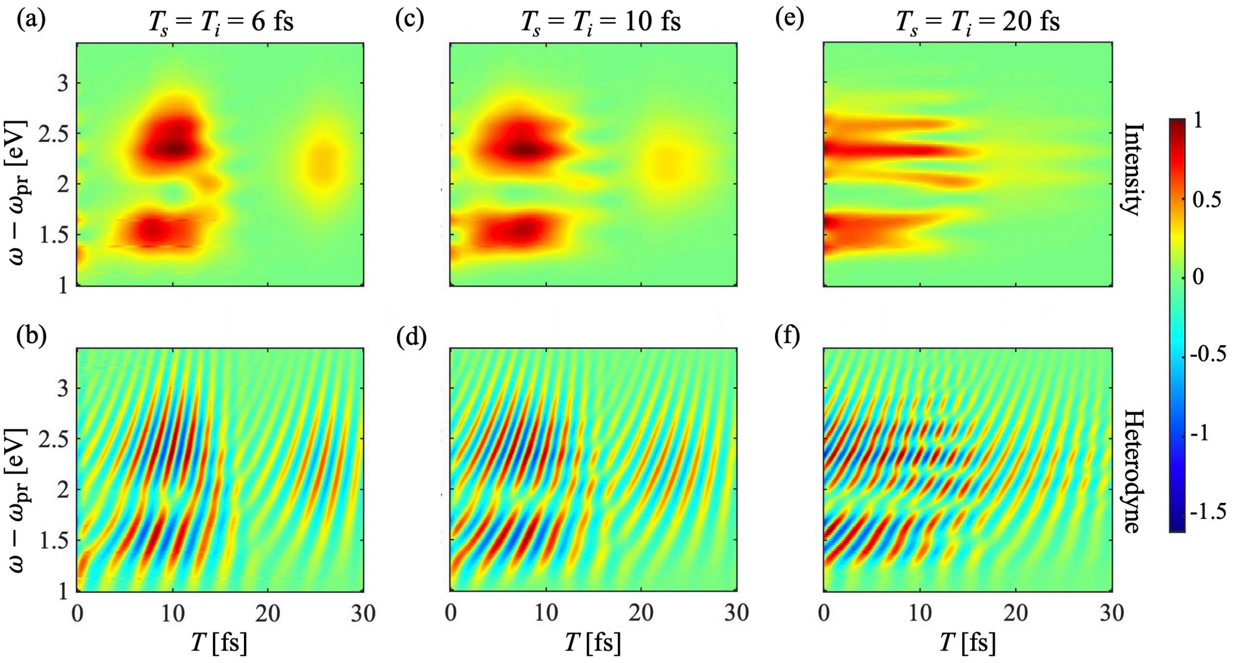

FIG. S3: (Top) Intensity-correlated signal QFTRS for time-evolving electronic coherence versus the delay T between entangled photons and pump pulse. (Bottom) Same for top row but for heterodyne detection. $\sigma_0 = 0.82$eV$(\sigma_0^{-1} = 5$fs) is the bandwidth of SPDC pump field and $T_s = T_i$ means the two photons arrive simultaneously. Parameters are taken from 4-oriented amino-4'nitrostilbene, i.e., $\omega_e = 7.1$eV, $\omega_{e_1} = 5.3$eV, $\omega_{e_2} = 5.7$eV, $v_h = 0.26$eV, $F_1 = 2.2$, $F_2 = 1.3$, $D_1^{-1/2} = 30$fs and $D_2^{-1/2} = 20$fs.

absorbing the frequency renormalization into $\tilde{\omega}_{e,e_j}$, where $e^{-F_j}F_j^n/n!$ is the Franck-Condon factor [12, 13].

With the coherence, we are able to find the Raman line-shape function

$$f_{e,e_j}(T) = 2\pi\int_{-\infty}^{\infty}\mathrm{d}\tau\rho_{e,e_j}(\tau)e^{i\omega(\tau-T)}\tilde{\Phi}(\tau-T,\omega_i) = 2\pi\sum_{n=0}^{\infty}\rho_{e,e_j}^{(n)}(T)g_{n,e_j}(\omega,T) \tag{S38}$$

where

$$\begin{aligned}\rho_{e,e_j}^{(n)}(T) &= \rho_{e,e_j}(0)\left(e^{-F_j}\frac{F_j^n}{n!}\right)e^{-i(\tilde{\omega}_{e,e_j}+nv_h)T-D_jT^2}\\ g_{n,e_j}(\omega,T) &= \int_{-\infty}^{\infty}\mathrm{d}\tau\ e^{i(\omega-\tilde{\omega}_{e,e_j}-nv_h)\tau}e^{-D_j(\tau^2+2T\tau)}\tilde{\Phi}(\tau,\omega_i).\end{aligned} \tag{S39}$$

and $\tilde{\Phi}(\tau,\omega_i) \equiv \frac{1}{2\pi}\int_{-\infty}^{\infty}\Phi(\omega,\omega_i)e^{-i\omega\tau}\mathrm{d}\omega$. Substituting Eq.(S38) into Eq.(S8) and (S28) we obtain the intensity-correlated and heterodyne-detected Raman signals

$$S_{\rm QE}(\omega,\omega_i;T) = \frac{N(N-1)}{8\pi^2}|\mathcal{E}_{s,\omega}|^6|\mathcal{E}_{i,\omega_i}|^2\left|\sum_{e_1}\sum_{n=0}^{\infty}\alpha_{e,e_1}^*g_{n,e_1}(\omega,T)\rho_{e,e_1}^{(n)}(T)\right|^2 \tag{S40a}$$

$$S_{\rm QEHD}(\omega,\omega_i;T) = 2N|\mathcal{E}_{s,\omega}|^2|\mathcal{E}_{i,\omega_i}|^2\sum_{e_1}\sum_{n=0}^{\infty}\mathrm{Im}\left[\alpha_{e,e_1}^*\Phi^*(\bar{\omega},\omega_i)g_{n,e_1}(\omega,T)\rho_{e,e_1}^{(n)}(T)\right] \tag{S40b}$$

Knowing the two-photon wave function one may be able to compute $g_{n,j}(\omega,T)$, so that the quantum Raman signals will be obtained. Alternatively, for the timescale of interest $T < 1/\sqrt{D_j}$, we can perform the integral in Eq.(S39) for arbitrary two-photon amplitude, such that

$$g_{n,e_j}(\omega,T) \simeq \int_{-\infty}^{\infty}\mathrm{d}\tau\ e^{[i(\omega-\tilde{\omega}_{e,e_j}-nv_h)-2D_jT]\tau}\tilde{\Phi}(\tau,\omega_i) = \Phi(\omega-\tilde{\omega}_{e,e_j}-nv_h+2iD_jT,\omega_i) \tag{S41}$$

which yields

$$S_{\rm QE}(\omega,\omega_i;T) \simeq \frac{N(N-1)}{8\pi^2}|\mathcal{E}_{s,\omega}|^6|\mathcal{E}_{i,\omega_i}|^2\left|\sum_{e_1}\sum_{n=0}^{\infty}\alpha^*_{e,e_1}\Phi(\omega-\tilde{\omega}_{e,e_1}-nv_h+2iD_jT,\omega_i)\rho^{(n)}_{e,e_1}(T)\right|^2 \tag{S42a}$$

$$S_{\rm QEHD}(\omega,\omega_i;T) \simeq 2N|\mathcal{E}_{s,\omega}|^2|\mathcal{E}_{i,\omega_i}|^2\sum_{e_1}\sum_{n=0}^{\infty}{\rm Im}\left[\alpha^*_{e,e_1}\Phi^*(\bar{\omega},\omega_i)\Phi(\omega-\tilde{\omega}_{e,e_1}-nv_h+2iD_jT,\omega_i)\rho^{(n)}_{e,e_1}(T)\right]. \tag{S42b}$$

Eq.(S42) indicates the multiple Raman resonance (lines) $\omega-\omega_{\rm pr}=\tilde{\omega}_{e,e_1}+nv_h$ in which the intensity is governed by the Franck-Condon factor quantifying the vibronic interaction. The spectral resolution is T_s^{-1} that may be greatly enhanced, as dictated by the two-photon amplitude. The two Raman signals given in Eq.(S42) are highly time- and frequency-resolved, revealing the ultrafast coherent dynamics of the electronically excited states.

Next we discuss the case of longer timescale that acquires a more precise calculation of the function $g_{n,j}(\omega,T)$. To that end, we consider the pump pulse of Lorentzian shape to create the two photon amplitude such that

$$\Phi(\omega_s,\omega_i) = \frac{A_0}{\omega_s+\omega_i-\omega_0+i\sigma_0}{\rm sinc}\left[(\omega_s+\omega_i-\omega_0)\frac{T_s}{2}\right]e^{i(\omega_s+\omega_i-\omega_0)T_s/2}. \tag{S43}$$

Inserting Eq.(S43) into Eq.(S39) some tedious algebra gives ($\omega_n\equiv\omega-\tilde{\omega}_{e,e_j}-nv_h$)

$$\begin{aligned} g_{n,e_j}(\omega,T) =& \frac{2\pi iA_0}{\sigma_0}\left[\int_0^1 {\rm d}x\ e^{-D_jT_s^2\left(x^2+\frac{2T}{T_s}x\right)}e^{i(\omega_n-\omega_{\rm pr})T_sx}\left(e^{-\sigma_0T_sx}-1\right)\right.\\ &\left.+\left(1-e^{\sigma_0T_s}\right)\int_1^{\infty}{\rm d}x\ e^{-D_jT_s^2\left(x^2+\frac{2T}{T_s}x\right)}e^{[i(\omega_n-\omega_{\rm pr})-\sigma_0]T_sx}\right] \end{aligned} \tag{S44}$$

and the integrals above can be carried out analytically using complementary error function ${\rm erfc}(z)=1-\frac{2}{\sqrt{\pi}}\int_0^z e^{-y^2}{\rm d}y$, so that the Raman signals can be illustrated from Eq.(S40b).

The role of photon entanglement can be elucidated by the comparison to the fully separable light such that $\Phi(\omega_s,\omega_i)=\Phi_s(\omega_s-\omega_0)\Phi_i(\omega_i-\omega_0)$ where

$$\Phi_s(\omega_s-\omega_0)=\frac{A_0}{\omega_s-\omega_0+i\sigma_0},\quad \Phi_i(\omega_i-\omega_0)=\frac{B_0}{\omega_i-\omega_0+i\sigma_0}. \tag{S45}$$

We then find the line-shape function

$$\begin{aligned} g_{n,e_j}(\omega,T) &= -iA_0\Phi_i(\omega_i-\omega_0)\int_0^{\infty}e^{[i(\omega_n-\omega_0)-\sigma_0]\tau}e^{-D_j(\tau^2+2T\tau)}{\rm d}\tau\\ &=\frac{A_0}{2i}\sqrt{\frac{\pi}{D_j}}e^{-\frac{(\omega_n-\omega_0+i(2D_jT+\sigma_0))^2}{4D_j}}{\rm erfc}\left(\frac{2D_jT+\sigma_0-i(\omega_n-\omega_0)}{2\sqrt{D_j}}\right)\Phi_i(\omega_i-\omega_0) \end{aligned} \tag{S46}$$

which may be much broader than $g_{n,e_j}(\omega,T)$ with the entangled photons in Eq.(S44) given $\sigma_0\gg T_s^{-1}$.

Fig.S3 illustrates the intensity-correlated and heterodyne-detected QFRS for various arrival times of entangled twin photons. Comparing the left, middle and right columns, more vibronic states associated with the coherence dynamics can be resolved when the two photons arrive later. This is in support of the spectral resolution T_s^{-1} claimed in the main text which reveals a great enhancement not accessible by classically shaped pulses.

IV. COHERENT RAMAN SIGNAL WITH CLASSICAL PULSES

With classical probe pulse, the excited-state Raman signal reads

$$S^{\rm (cl)}_{\rm HM}(\omega,T)=N(N-1)|\mathcal{E}_\omega|^4\left|\sum_j\alpha^*_{e,e_j}f^{\rm (c)}_{e,e_j}(T)\right|^2 \tag{S47a}$$

$$S^{\rm (cl)}_{\rm HD}(\omega,T)=N|\mathcal{E}_\omega|^2\sum_j{\rm Im}\left[\alpha^*_{e,e_j}E^*_{\rm LO}(\bar{\omega})f^{\rm (c)}_{e,e_j}(T)\right] \tag{S47b}$$

where the Raman line-shape function is

$$f^{(c)}_{e,e_j}(T) = \frac{2\pi E_0 \sigma_0}{\sqrt{\sigma_0^2 + 2D_j}} \sum_{n=0}^{\infty} \rho^{(n)}_{e,e_j}(T)\, e^{-\frac{\left(\omega - \omega_0 - \tilde{\omega}_{e,e_j} - n v_h + 2iD_j T\right)^2}{2(\sigma_0^2 + 2D_j)}} \tag{S48}$$

for the Gaussian pulse shape $E(\omega - \omega_0) = E_0 e^{-(\omega-\omega_0)^2/2\sigma_0^2}$.

---

[1] Petrov, G. I. *et al.* Comparison of coherent and spontaneous Raman microspectroscopies for noninvasive detection of single bacterial endospores. *Proceedings of the National Academy of Sciences of the United States of America* **104**, 7776-7779 (2007).

[2] Traverso, A. J. *et al.* Two-photon infrared resonance can enhance coherent Raman scattering. *Physical Review Letters* **120**, 063602-063606 (2018).

[3] Dorfman, K. E., Schlawin, F. & Mukamel, S. Stimulated Raman spectroscopy with entangled light: enhanced resolution and pathway selection. *The Journal of Physical Chemistry Letters* **5**, 2843-2849 (2014).

[4] Kowalewski, M. *et al.* Catching conical intersections in the act: monitoring transient electronic coherences by attosecond stimulated X-ray Raman signals. *Physical Review Letters* **115**, 193003-193007 (2015).

[5] Zhang, Z. *et al.* Utilizing microcavities to suppress third-order cascades in fifth-order Raman spectra. *The Journal of Physical Chemistry Letters* **8**, 3387-3391 (2017).

[6] Mukamel, S. Principles of Nonlinear Optical Spectroscopy (New York: Oxford University Press, 1999).

[7] Scully, M. O. *et al.* FAST CARS: engineering a laser spectroscopic technique for rapid identification of bacterial spores. *Proceedings of the National Academy of Sciences of the United States of America* **99**, 10994-11001 (2002).

[8] Dorfman, K. E., Schlawin, F. & Mukamel, S. Nonlinear optical signals and spectroscopy with quantum light. *Reviews of Modern Physics* **88**, 045008-045074 (2016).

[9] Law, C. K., Walmsley, I. A. & Eberly, J. H. Continuous frequency entanglement: effective finite Hilbert space and entropy control. *Physical Review Letters* **84**, 5304-5307 (2000).

[10] Yan, Y. J. & Mukamel, S. Femtosecond pump-probe spectroscopy of polyatomic molecules in condensed phases. *Physical Review A* **41**, 6485-6504 (1990).

[11] Barbara, P. F., Meyer, T. J. & Ratner, M. A. Contemporary issues in electron transfer research. *The Journal of Physical Chemistry* **100**, 13148-13168 (1996).

[12] Franck, P. J. & Dymond, E. G. Elementary processes of photochemical reactions. *Transactions of the Faraday Society* **21**, 536-542 (1926).

[13] Huang, K. & Rhys, A. Theory of light absorption and non-radiative transitions in *F*-centres. *Proceedings of the Royal Society A: Mathematical, Physical and Engineering Sciences* **204**, 406-423 (1950).
